# Enhanced Immunomodulation in Inflammatory Environments Favors Human Cardiac Mesenchymal Stromal-Like Cells for Allogeneic Cell Therapies

**DOI:** 10.3389/fimmu.2019.01716

**Published:** 2019-07-23

**Authors:** Falk Diedrichs, Meaghan Stolk, Karsten Jürchott, Marion Haag, Michael Sittinger, Martina Seifert

**Affiliations:** ^1^Berlin Institute of Health (BIH), Berlin, Germany; ^2^BIH Center for Regenerative Therapies (BCRT), Charité-Universitätsmedizin Berlin, Corporate Member of Freie Universität Berlin, Humboldt-Universität zu Berlin, and Berlin Institute of Health, Berlin, Germany; ^3^Institute of Medical Immunology, Charité-Universitätsmedizin Berlin, Corporate Member of Freie Universität Berlin, Humboldt-Universität zu Berlin, and Berlin Institute of Health, Berlin, Germany; ^4^Tissue Engineering Laboratory, Charité-Universitätsmedizin Berlin, Corporate Member of Freie Universität Berlin, Humboldt-Universität zu Berlin, and Berlin Institute of Health, Berlin, Germany

**Keywords:** cardiac-derived cells, immunogenicity, immunomodulation, inflammation, IFNγ, IDO

## Abstract

Rising numbers of patients with cardiovascular diseases and limited availability of donor hearts require new and improved therapy strategies. Human atrial appendage-derived cells (hAACs) are promising candidates for an allogeneic cell-based treatment. In this study, we evaluated their inductive and modulatory capacity regarding immune responses and underlying key mechanisms *in vitro*. For this, cryopreserved hAACs were either cultured in the presence of interferon-gamma (IFNγ) or left unstimulated. The expression of characteristic mesenchymal stromal cell markers (CD29, CD44, CD73, CD105, CD166) was revealed by flow cytometry that also highlighted a predominant negativity for CD90. A low immunogeneic phenotype in an inflammatory milieu was shown by lacking expression of co-stimulatory molecules and upregulation of the inhibitory ligands PD-L1 and PD-L2, despite *de novo* expression of HLA-DR. Co-cultures of hAACs with allogeneic peripheral blood mononuclear cells, proved their low immunogeneic state by absence of induced T cell proliferation and activation. Additionally, elevated levels of IL-1β, IL-33, and IL-10 were detectable in those cell culture supernatants. Furthermore, the immunomodulatory potential of hAACs was assessed in co-cultures with αCD3/αCD28-activated peripheral blood mononuclear cells. Here, a strong inhibition of T cell proliferation and reduction of pro-inflammatory cytokines (IFNγ, TNFα, TNFβ, IL-17A, IL-2) were observable after pre-stimulation of hAACs with IFNγ. Transwell experiments confirmed that mostly soluble factors are responsible for these suppressive effects. We were able to identify indolamin-2,3-dioxygenase (IDO) as a potential key player through a genome-wide gene expression analysis and could demonstrate its involvement in the observed immunological responses. While the application of blocking antibodies against both PD-1 ligands did not affect the immunomodulation by hAACs, 1-methyl-L-tryptophan as specific inhibitor of IDO was able to restore proliferation and to lower apoptosis of T cells. In conclusion, hAACs represent a cardiac-derived mesenchymal stromal-like cell type with a high potential for the application in an allogeneic setting, since they do not trigger T cell responses and even increase their immunomodulatory potential in inflammatory environments.

## Introduction

Cardiovascular diseases are the leading cause of morbidity and mortality worldwide with ischemic heart disease alone being responsible for almost 1.8 million deaths per year in Europe (20% of all deaths; European Heart Network, 2017^1^). Even though there is a range of existing therapeutic strategies available, which have beneficial effects on the improvement of life quality and the extension of lifespan in cardiovascular patients, they often leave them with no other causal therapy option than heart transplantation (1, 2).

Numerous attempts using a variety of different cell sources were initiated over the last 20 years for development of new therapeutic treatments to induce cardiac regeneration (3). Particularly autologous cell sources, ranging from hematopoietic cells (4), over mesenchymal stromal cells (MSCs) from different tissues (5–7), to various cardiac progenitor cells (CPCs) (8–11) have been heavily investigated in this context. Specifically, the use of CPCs led to promising results: animal models of myocardial infarction demonstrated improved cardiac function after cell transplantation (12) and even first clinical studies in humans (SCIPIO and CADUCEUS trials) were able to show moderately increased regeneration of cardiac tissue (13–16).

An alternative mesenchymal-like cardiac cell type for an autologous therapeutic application in heart injury, are so called cardiac-derived adherent proliferating cells (CardAPs). This unique cell type derived from endomyocardial biopsies shares typical characteristics with MSCs but clearly distinguishes itself from all other cell types used so far in cell therapeutic application. CardAPs are positive for CD44, CD73, CD105, and CD166 but express neither the hematopoietic markers CD14, CD34 and are strikingly low for the marker CD90, which is otherwise characteristic for MSCs and fibroblasts (17). These cardiac-derived cells demonstrated increased regenerative potency by mediating angiogenesis and cardiomyogenesis, reducing cardiac hypertrophy and exhibiting immunomodulatory capacities to induce an anti-inflammatory environment (18–20). Our own immunological *in vitro* tests with these mesenchymal-like CardAPs proved their low immunogeneic status as well as the capacity to modulate the immune system toward an anti-inflammatory state (21). However, recent clinical phase-I studies with mesenchymal cell types highlighted some of the fundamental limitations of autologous cell sources (22). Manufacturing a sufficient amount of a patient-specific cell product is time consuming, thus preventing immediate availability in acute situations. Additionally, harvesting from elderly diseased patients with co-morbidities raised further concerns regarding the functional integrity and overall survival of obtained cells (23). Furthermore, it is the recent scientific consensus that every stromal cell source has to be considered as an independent entity and requires a comprehensive phenotypical and functional characterization using standardized protocols, with a particular focus on their immunological properties and immunomodulatory potency (24). This would help to identify an adequate cell source or cell subset and to promote the appropriate and safe application as a cell therapeutic or even as cell free products based on paracrine released vesicles or mediators.

For that reason, it is essential to evaluate the potential use of allogeneic cardiac-derived cells, since they can be harvested from healthy donors, have the benefit of being available at any time and can be assessed and manipulated in advance to fit the patient's needs (25). This might be important, since the transplantation of allogeneic cells or tissues always poses the risk of recognition by the recipient's immune system and induction of unwanted inflammatory responses by secretion of allo-antibodies (26, 27) or even T cell-mediated rejection responses (28, 29).

Experimental data by others with a cardiac-derived mesenchymal-like cell type indicated that those cryopreserved c-Kit^+^ CPCs displaying low immunogeneic properties, were able to reduce local inflammatory processes and limit T cell proliferation in already ongoing immunoreactions *in vitro* (30). Additionally, the phase-I/-II CAREMI trial already proved the principal safety of allogeneic cell transplantation with previously mentioned c-Kit^+^ selected CPCs by absence of major adverse effects after intracoronary injection (31). However, the overall benefit in cardiac improvement remains ambiguous and demands the evaluation of additional allogeneic cell sources.

Our group recently described the atrial appendage as a potential new cell source for human atrial appendage-derived cells (hAACs) that are a CD90^low^ cell product with similar pro-angiogenic characteristics compared to the endomyocardial-derived CardAPs (32). hAACs can be easily isolated from cardiac tissue and would allow allogeneic treatment for a substantial number of patients. These cells represent a mesenchymal-like cardiac-derived cell type based on the expression of the characteristic markers CD29, CD44, CD73, CD105, and CD166, but predominantly lack expression of CD90 at the same time. Precisely, this CD90^low^ phenotype could provide a beneficial tool for the enhanced repair capacity of a cell product, since it was shown that CD90 expression on cardiosphere-derived cells is negatively correlated with the scar size of injured heart tissue after cell application in myocardial infarction (33). In addition, first studies with hAACs in a mouse model of Coxsackievirus B3 (CVB3)-induced myocarditis could demonstrate, that intravenous application was able to improve the left ventricular heart function and contractility as well as to decrease tissue collagen I expression. In this experimental mouse study, immunomodulatory effects were also confirmed by detecting reduced levels of TGFβ-producing CD68^+^ cells and regulatory T cells in the spleen of treated animals (34).

To ensure the safety and efficacy of this new hAAC product for an allogeneic transplantation in humans, it is crucial to determine whether these cells trigger immune responses in an inflammatory scenario, as seen in cell transplantation. Therefore, we aimed to assess the immunological properties of this defined cell product, test their interaction with cells from the adaptive immune system *in vitro* and gain insights into the underlying mechanism of action. First, we confirmed a mesenchymal-like surface marker expression profile after cryopreservation and assessed changes of the immune phenotype under inflammatory conditions. Second, hAACs were evaluated in immune cell co-cultures to study potential immunogeneic properties and the capacity to modulate adaptive immune responses. We could identify several potential molecules explaining the observed immune modulatory effects by a genome-wide gene expression analysis. Finally, our data revealed that indolamine-2,3-dioxygenase (IDO) is a key player of the immunomodulation by hAACs mediating the inhibition of T cell proliferation and the induction of their apoptosis.

## Materials and Methods

### Isolation and Culture of Human Atrial Appendage-Derived Cells (hAACs)

Right atrial appendages, that were obtained during open-heart surgery at Deutsches Herzzentrum Berlin from eight patients, were used to generate hAACs as previously described (32). Briefly, the right atrial appendages were reduced to fragments of 1 mm3 and cultured in Iscove's Modified Dulbecco's Medium (IMDM; Biochrom, Berlin, Germany) containing 10% allogeneic human serum (German Red Cross, Berlin, Germany), 100 U/mL penicillin and 100 μg/mL streptomycin (both from Biochrom). Outgrowing cells were harvested after about 13 days with 0.05% trypsin/0.02% EDTA (Biochrom) and then subjected to immunomagnetic sorting with CD90 microbeads (MACS; human CD90 MicroBeads kit, Miltenyi Biotec, Bergisch Gladbach, Germany). The resulting CD90^low^ cell population was grown under standard culture conditions (37°C in 21% O_2_ and 5% CO_2_ atmosphere) at a density of 6000 cells/cm^2^ in complete medium (cIDH) consisting of equal amounts of IMDM/DMEM/Ham's F12 (IDH; all Biochrom) and supplemented with 5% male heat-inactivated human AB serum (Sigma-Aldrich, St. Louis, MO, USA), 100 U/mL penicillin and 100 μg/mL streptomycin (Gibco® Life Technologies, Grand Island, NY, USA), 20 ng/mL basic fibroblast growth factor and 10 ng/mL epithelial growth factor (both from Preprotech, Hamburg, Germany) for further expansion of the purified cell product. Subsequently, hAACs were cryopreserved for at least 6 months to mimic conditions of a cell bank. After thawing, cells were routinely passaged once in cIDH medium before performing assays and were used between passages 2 and 8. Tissues were obtained according to the local guidelines of the Charité - Universitätsmedizin Berlin as well as the Declaration of Helsinki and the study was approved by the ethics committee of the Charité - Universitätsmedizin Berlin (No. 4/028/12). Human leukocyte antigen (HLA)-typing of the cells were performed in the HLA-Laboratory of the Charité - Universitätsmedizin Berlin by SSO-PCR (low) for HLA-A, HLA-B and HLA-DR. A list of all HLA-typed cells is available in the Supplementary Table 1.

### Culture of Human Umbilical Cord-Derived Mesenchymal Stromal Cells (MSCs) and Umbilical Vein Endothelial Cells (HUVECs)

Due to their known immunomodulatory potential as previously described (35), human umbilical cord-derived MSCs were used as control cells in the immune cell co-culture experiments. Cells were kindly provided by Dirk Strunk's laboratory at the Institute of Experimental and Clinical Cell Therapy and Spinal Cord & Tissue Regeneration Center, Paracelsus Medical University (PMU) Salzburg, Austria and were obtained for human cell and tissue sample collection from the Institutional Review Board of the Medical University of Graz (protocol 19-252 ex 07/08) as described (36). Umbilical cord samples were collected from mothers that gave written informed consent after full-term pregnancies in accordance with the Declaration of Helsinki. After thawing, MSCs were grown in alpha-modified minimum essential medium (alpha-MEM; Biochrom), supplemented with 5% human male heat-inactivated AB serum (Sigma-Aldrich), 2 mM L-glutamine, 100 U/mL penicillin and 100 μg/mL streptomycin (both from Gibco® Life Technologies) at 37°C in 21% O_2_ and 5% CO_2_ atmosphere. HLA-Typing of the donor cells was performed by SSP PCR using Olerup SSPTM low-resolution kits (GenoVision Inc., Philadelphia, PA, USA).

HUVECs were used as positive controls in the immune cell co-culture experiments (Cascade Biologics®, Thermo Fisher Scientific, Rochester, NY, USA and Lonza, Wakersville, MD, USA). After thawing, HUVECs were cultured in EGM-2 (Lonza) with 5% human male heat-inactivated AB serum (Sigma-Aldrich), 100 U/mL penicillin and 100 μg/mL streptomycin (Gibco® Life Technologies) for further expansion.

Both cell types were passaged once before performing assays and were used between passages 2 and 8.

### PBMC Isolation

Human peripheral blood mononuclear cells (PBMCs) were isolated from buffy coats (German Red Cross, Berlin, Germany; approved by the local Ethical Committee, EA1/226/14) by using a Biocoll gradient (Biochrom), as previously described (37). Briefly, following centrifugation at 800 g for 30 min without brake, PBMCs were harvested from the interphase and were washed three times with cold phosphate buffered saline solution (PBS; Biochrom). Cells were cryopreserved for later experimental use in liquid nitrogen. HLA-typing was performed in the HLA-Laboratory of the Charité - Universitätsmedizin Berlin by SSO-PCR (low) for HLA-A, HLA-B and HLA-DR.

### Immunocytochemistry Analysis of hAACs

hAACs were plated on collagen I-coated (BD Biosciences, San Jose, CA, USA) 24 well dishes (Falcon, BD Biosciences). After incubation overnight, wells were washed three times with Hank's Balanced Salt Solution (HBSS; Gibco® Life Technologies) containing Mg^2+^ and Ca^2+^, fixed with 4% paraformaldehyde (PFA; Roth, Karlsruhe, Germany) for 10 min at room temperature and washed twice with HBSS. Subsequently, the cells were incubated with 5 μg/mL wheat germ agglutinin (WGA; Biotium, Fremont, CA, USA) for 10 min at 37°C. After washing twice with HBSS, nuclei were counterstained for 15 min at room temperature with 4,6-diamidino-2-phenylindole (DAPI; Molecular probes™, Thermo Fisher). Images were taken with an Operetta® High Content Imaging System and image analysis performed by the Columbus™ Image Data Storage and Analysis System (both from Perkin Elmer, Waltham, MA, USA).

### Fluorescence Staining of Cells and Flow Cytometry (FACS)

Non-adherent PBMCs were resuspended by pipetting, and adherent hAACs were first harvested using a 0.05% trypsin solution with EDTA (Gibco® Life Technologies) and transferred to 5 mL FACS tubes (Falcon, BD Biosciences). Staining procedure was performed as previously described (37). Briefly, cells were washed once with cold PBS, resuspended in a final volume of 50 μL antibody mix in cold FACS buffer [PBS supplemented with 1% fetal calve serum (FCS; both Biochrom)] and incubated for 30 min at 4°C in the dark. A list of all used antibodies and dyes as well as the respective dilution is available in the online Supplementary Table 2. Antibody mixes also contained the Live/Dead® violet Staining Kit (Molecular probes™, Thermo Fisher Scientific) in order to exclude dead cells from the analysis. After antibody incubation, the samples were washed with cold FACS buffer and resuspended in 1% PFA (Roth, Karlsruhe, Germany) in FACS buffer. Samples were kept at 4°C in the dark until measurement on a FACS Canto II device with FACS Diva software (Becton Dickinson, San Jose, CA, USA). Data analysis was performed using FlowJo software (TreeStar Inc., Ashland, OR, USA; RRID:SCR_008520). Gating strategies for the FACS-analysis of hAACs and PBMCs are shown in Supplementary Figure 1. Expression of a marker is presented either as percentage of positive cells against the unlabeled control or as geometric mean of fluorescence intensity (MFI).

### Kinetic Analysis of hAAC Surface Marker Expression

hAACs were seeded on 24 well-plates (Costar®, Corning Incorporated, Kennebunk, ME, USA) at a density of 3 × 10^5^ cells and were cultured in cIDH medium overnight. Afterwards, hAACs were either directly harvested for evaluation of constitutive MSC marker expression (CD90, CD29, CD44, CD73, CD105, CD166, CD14, CD31, CD45, c-Kit) or stimulated with 100 ng/mL of interferon-gamma (IFNγ) or a combination of 100 ng/mL IFNγ and 100 ng/mL tumor necrosis factor alpha (TNFα; both from Miltenyi Biotec) for evaluation of the immunological (HLA-ABC, HLA-E, HLA-DR, CD80, CD86, PD-L1 and PD-L2) and characteristic MSC markers (CD90, CD29, CD44, CD73, CD166). hAACs were stimulated and harvested after one, 2 and 5 days, respectively for flow cytometric analysis as described before.

### hAAC/Immune Cell Co-cultures

hAACs from six different donors and control cultures with MSCs and HUVECs were seeded on rat tail collagen I-coated (BD Biosciences) 24 well plates (Costar®, Corning Incorporated) at a density of 2 × 10^5^ cells. After attachment overnight, the adherent cells were either stimulated with 100 ng/mL IFNγ (Miltenyi Biotec) or left unstimulated for 48 h. Afterwards, the confluent monolayers were irradiated with 60 Gray using a gamma-radiation source (GSM GmbH, Leipzig, Germany) to maintain a stable cell number throughout the assay. Human HLA-mismatched PBMCs were thawed, washed three times with cold PBS (Biochrom) and labeled with 5 μM carboxyfluorescein succinimidyl ester (CFSE; Biolegend, San Diego, CA, USA) for 3 min. The staining reaction was then stopped by incubating with cold heat-inactivated human AB serum (Sigma-Aldrich) for 1 min. After washing three times with cold PBS, 3 × 10^5^ CFSE-labeled PBMCs, that were a complete mismatch to the respective hAAC donor, were added to the hAAC, MSC and HUVEC cultures. The resulting co-cultures were maintained in 1 mL of very low endotoxin (VLE)-Roswell Park Memorial Institute (RPMI; Biochrom), supplemented with 10% human male heat-inactivated AB serum (Sigma-Aldrich), 100x L-glutamine solution, 100 U/mL penicillin and 100 μg/mL streptomycin (all from Gibco® Life Technologies). After 4 days, 250 μL of co-culture supernatant were taken for cytokine detection and 750 μL of completely supplemented VLE-RPMI were added to the cultures. Following seven days of incubation, PBMCs were harvested, stained for human immune cell defining surface markers and analyzed by flow cytometry.

### Proliferation Based Immunomodulation Assay

Analogous to the hAAC/immune cell co-culture analysis, hAACs, MSCs and HUVECs were cultured on rat tail collagen I-coated (BD Biosciences) 24 well plates (Costar®, Corning Incorporated) at a density of 2 × 10^5^ cells in the presence or absence of 100 ng/mL IFNγ (Miltenyi Biotec) for 48 h. Human PBMCs were CFSE-labeled as mentioned before and activated with a combination of 0.02 μg/mL anti-CD3 (OKT3 antibody, Janssen-Cilag, Neuss, Germany) and 0.03 μg/mL anti-CD28 (BD Biosciences). Lastly, 1 × 10^6^ PBMCs were added to the cultures in 2 mL of completely supplemented VLE-RPMI medium. After 3 days, supernatants were taken for cytokine detection and PBMCs were harvested, stained for human immune cell defining surface markers and analyzed by flow cytometry.

Experimental settings were repeated under transwell conditions. Here, hAACs were seeded at a density of 4 × 10^4^ cells at the bottom of rat tail collagen I-coated 24 well plates. After stimulation with IFNγ, polycarbonate transwell inserts with 0.4 μm pore size (Costar®, Corning Incorporated) were initially equilibrated for 1 h at 37°C with RPMI and subsequently 2 × 10^5^ CFSE-labeled PBMCs were seeded into the inserts. After a co-culture time of 3 days at 37°C in a 21% O_2_ and 5% CO_2_ atmosphere, PBMC were harvested for flow cytometric analysis of proliferation and surface marker expression.

To selectively analyze the effects of either indoleamin-2,3-dioxygenase (IDO) or both programmed death-1 (PD-1) ligands (PD-L1 and PD-L2) in immune cell co-cultures with hAACs, 1 mM 1-methyl-L-tryptophan (1-MT; Sigma-Aldrich) was provided 2 h prior to addition of CFSE-labeled or unlabeled PBMCs and 5 μg/mL of purified anti-PD-L1 and anti-PD-L2 antibodies (both Biolegend) were added 12 h before CFSE-labeled or unlabeled PBMCs were added to the hAAC cultures.

### Cytokine Detection Assays

Supernatants of mono- and co-cultures of hAACs, MSCs and HUVECs from the proliferation induction experiments were tested for IL-1β, IFNα, IFNγ, TNFα, MCP-1, IL-6, IL-8, IL-10, IL-12p70, IL-17A, IL-18, IL-23, and IL-33 using the Legendplex™ human inflammation 13-plex panel (Biolegend). The minimum detectable concentration of each cytokine is given as 0.6–2.1 pg/mL. Samples were treated following manufacturer's instructions and measured with a FACS Canto II device (Becton Dickinson).

Supernatants of hAAC co-cultures from the direct-contact immune modulation experiments were analyzed for their content of IL-1β, IL-2, IL-5, IL-10, IL-13, IL-17A, TNFα, TNFβ, IFNγ, and MDC by a multiplex assay using a Milliplex® human multi-analyte Luminex® kit (Merck KGaA, Darmstadt, Germany). Samples were treated following manufacturer's instructions and measured with a Bio-Plex® 200 multiplex analysis device (Bio-Rad®, California, USA).

### Genome-Wide Gene Expression Profile

Human GeneChip U133 Plus 2.0 (Affymetrix, Santa Clara, CA, USA) was used for genome-wide gene expression profiling of hAAC samples covering over 47,000 transcripts (54,765 probes in total including double entries). RNA samples of unstimulated and IFNγ pre-stimulated hAACs were prepared with GeneChip® 3′ IVT Express Kit and GeneChip® Hybridisation, Wash and Stain Kit (Affymetrix) according to the manufacturer's instructions. In brief, 250 ng total RNA was used for cDNA synthesis and subsequent *in vitro* transcription (IVT) to amplified RNA (aRNA). 12.5 μg fragmented aRNA was used for hybridization on the chip for 16 h at 45°C. Finally, the chips were washed, stained and scanned using the Affymetrix Gene Chip Scanner 3000. Affymetrix GeneChip Operating Software (GCOS) 1.4 was used to generate CEL data files, for raw data processing and for calculation of signal intensity, signal log ratio (SLR) and *p*-value of pairwise chip comparisons AF/NP. Quality control and pre-processing was done in R^2^ with the package “affy” (38). Raw data were normalized and log2-transformed using Robust Multi-array Average (RMA) algorithm implemented in this package. Thousand probe sets with the highest variances were selected in order to run a principle component analysis. Differentially expressed probe sets between the two treatment groups were selected by fitting linear models to the data and Bayesian statistics were run as implemented in the package “limma” (39). False discovery rates were used to adjust raw *p*-values for multiple testing and a minimal absolute log2-Foldchange of 1 was used for probe set selection. Mapping of differentially expressed probesets to genes and functional annotations of the DAVID database (40, 41) was done using the package “clusterProfiler” (42). Over-representation of differentially expressed genes in terms of the category “Biological Process” of the gene ontology system was done using the enrichDAVID()-function of this package. The eight top ranking results of this analysis were shown as GOcirc-plot using the “GOplot”-package (43).

### RNA Extraction, cDNA Synthesis and Quantitative Polymerase Chain Reaction (qPCR)

Total RNA was extracted from unstimulated and IFNγ pre-stimulated hAACs following 48 h of incubation using the RNeasy® Mini Kit (QIAGEN, Venlo, Netherlands) according to the manufacturer's protocol. After measuring the RNA concentration with the NanoDrop 2000 spectrophotometer (Thermo Fisher Scientific), cDNA was synthesized. The reverse transcription reaction was performed using TaqMan™ Reverse Transcription Reagents Kit (Invitrogen™, Thermo Fisher Scientific). Briefly, the following components were combined to perform a 20 μL reaction volume: nuclease-free water plus total RNA (1000 ng/μL), RNase inhibitor (20 U/μL), Mg_2_Cl, 10x RT Buffer, Random Hexamer Primer Mix (50 μM), dNTP Mixture (2.5 mM each dNTP) and Reverse Transcriptase (RT; 50 U/μL). Samples were incubated for 30 min at 48°C, 5 min at 95°C and subsequently cooled down at 4°C with a Thermo Flex Cycler Block (Analytik Jena, Jena, Germany). After the RT-PCR the concentration of the generated cDNA was measured with the NanoDrop 2000 to ensure a functional template for the subsequent qPCR. The qPCR was performed on a QuantStudio 6 Flex Real-Time PCR machine (Applied Biosystems, Thermo Fisher Scientific) using the SensiMix™ SYBR No-ROX kit (Bioline, London, UK). The thermal cycling conditions were comprised of a 95°C initial template denaturation for 20 s, followed by 40 cycles of PCR by applying 95°C for 15 s and 60°C for 20 s. Lastly, a final melt curve stage with 40 cycles comprising of 95°C for 15 s, 60°C for 60 s and 95°C for 15 s was performed. Three technical replicates of each sample were analyzed for gene expression of *IDO1, LGALS9, TLR3, PD-L1, PD-L2, PTGS1, HLA-G*, and *VCAM1*. All of the used primer sequences are listed in Table 1. The samples were normalized to the expression of the house keeping gene *HPRT* and data were analyzed using the delta-delta Ct (ΔΔCt) method. The final results are therefore calculated as fold change of target gene expression in IFNγ pre-stimulated hAAC samples relative to the unstimulated hAAC reference samples to demonstrate upregulation of differentially expressed genes.

**Table 1:** The Primer sequences of selected immunomodulatory genes.

**Gene**	**Forward primer (5**′** → 3**′**)**	**Reverse primer (3**′** → 5**′**)**
IDO1	CGGTCTGGTGTATGAAGG	CTAATGAGCACAGGAAGTTC
LGALS9	CACACATGCCTTTCCAGAAG	AAGAGGATCCCGTTCACCAT
TLR3	ATCTGTCTCATAATGGCTT	AGAAAGTTGTATTGCTGGT
PD-L1	GGCATCCAAGATACAAACTCAA	CAGAAGTTCCAATGCTGGATTA
PD-L2	GAGCTGTGGCAAGTCCTCAT	GCAATTCCAGGCTCAACATTA
PTGS1	TGTTCGGTGTCCAGTTCCAATA	ACCTTGAAGGAGTCAGGCATGAG
HLA-G	TTGGGAAGAGGAGACACGGAACA	AGGTCGCAGCCAATCATCCAC
VCAM1	CGTCTTGGTCAGCCCTTCCT	ACATTCATATACTCCCGCATCCTTC
HPRT	AGTCTGGCTTATATCCAACACTTC	GACTTTGCTTTCCTTGGTCAGG

### Raman Trapping Microscopy

Raman spectral acquisition was conducted using a BioRam® system (CellTool GmbH, Tutzing, Germany) equipped with an excitation laser wavelength of 785 nm and a laser power of 80 mW. The laser was focused through a 60x (NA 0.7) air objective. In all samples, 500 single cells were randomly selected under bright-field illumination and pinpointed for automatic spectra retrieval. Raman spectra were taken from the cytoplasm using accumulated scans of 3 × 10 s. Together with the spectra the x-y-z coordinates as well as bright-field images of each measured cell were stored. As control, 10 background measurements were taken from each sample. To assess acquired Raman spectra, multivariate data analysis was performed. Principal Component Analysis (PCA) was used for visualizing the datasets. PCA was implemented in Python 2.7, using the scikit-learn package (44). PCA score plots were used to look for clusters among the data. Circles in 2D scores plot depict 95% confidence intervals.

### Statistical Analysis

Statistical Analysis and graph generation was performed with GraphPad Prism 8.0 (Graphpad Software, La Jolla, USA; RRID:SCR_002798). Statistical analyses were chosen that do rely on non-parametric distribution, since all data sets were *n* ≤ 10. Statistical differences between two groups with only one variable were analyzed using the Mann-Whitney non-parametric *t*-test. For more than two groups with multiple variables, Kruskal Wallis one-way analysis of variance (ANOVA) with Dunn's post tests were applied. Statistical differences between two or more groups with more than two variables were analyzed using an ordinary two-way ANOVA with the Sidak's post-test. All results are shown as mean ± SEM and asterisks were assigned to the *p*-values in the following order: ^*^p ≤ *0.05;* ^**^ *p* ≤ *0.01;* ^***^p ≤ *0.001;* ^****^p ≤ *0.0001*.

## Results

### Cryopreserved hAACs Show Typical Characteristics for Cells of Mesenchymal Origin

**Figure 1:**
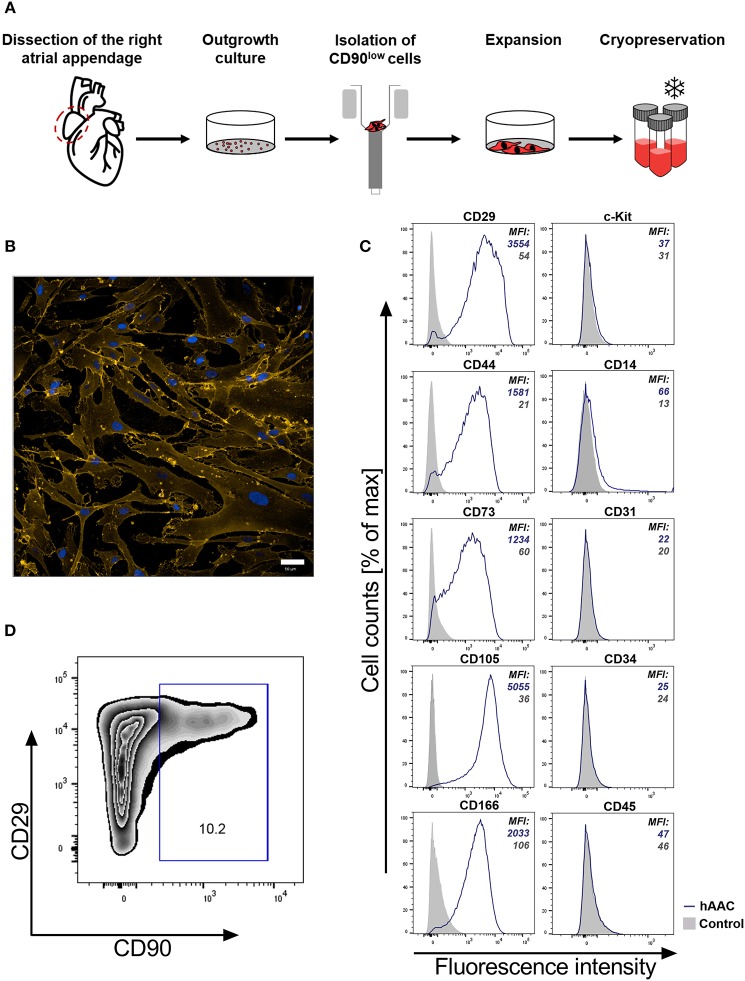
Characteristics of human atrial appendage-derived cells (hAAC). **(A)** The general procedure to generate hAACs is shown: right atrial appendages were harvested from patients during open-heart surgery. The dissected tissue was minced into small pieces and cultured. Subsequently, the fragment‘s outgrowth was harvested, immuno-magnetically sorted as CD90^low^ cells and seeded again for further expansion of the desired purified hAAC product. The cells were cryopreserved and long-term stored for later experimental use. **(B)** Representative immunofluorescence images show the characteristic morphology of one hAAC donor in a cell culture plate after 12 h of incubation. Cell membranes (orange) were stained with wheat germ agglutinin (WGA) and nuclei (blue) with 4,6-diamidin-2-phenylindol (DAPI). Scale bar represents 50 μm. **(C)** Generated hAACs were harvested by treatment with trypsin after passage four and stained with human-specific antibodies against surface markers characteristic for cells of mesenchymal origin (CD29, CD44, CD73, CD105, CD166), markers for exclusion of hematopoietic contaminants (CD14, CD31, CD34, CD45) and the cardiac progenitor marker c-Kit (CD117). Representative flow cytometry histograms of one hAAC donor are shown as fluorescence intensity against cell counts for all tested markers, indicating the mean of fluorescence intensity (MFI) of positive cells (blue) compared to the unlabeled controls (gray) for each marker. **(D)** The percentage of remaining CD90^+^ cells in the hAAC cell product is shown for one representative donor in a dot plot of CD90 against CD29.

It is crucial for a potential allogeneic application of hAACs to determine the general characteristics of these cells after long-term cryopreservation. hAACs from eight right atrial appendages were generated from outgrowth cultures by negative immunoselection for CD90, expanded in cell culture and cryopreserved for at least 6 months (Figure 1A). Thawed cells after 24 h in cell culture showed their distinctive morphology with long elongated cell bodies and fibroblast-like appearance (Figure 1B). Flow cytometry analysis for characteristic surface markers of mesenchymal cells confirmed the distinguishing marker profile of these cells. The hAAC cell product expressed most of the known mesenchymal stem- and progenitor markers (e.g., CD29, CD44, CD73, CD105, and CD166), while lacking the expression of the endothelial marker CD31, the hematopoietic markers CD14, CD34, and CD45 as well as the cardiac progenitor marker c-Kit (CD117) (Figure 1C). Yet, in contrast to classical fibroblasts and mesenchymal stromal cells, only a small proportion of cells was positive for CD90 (Figure 1D).

### Inflammatory Priming Alters the Immune Phenotype of hAACs

**Figure 2:**
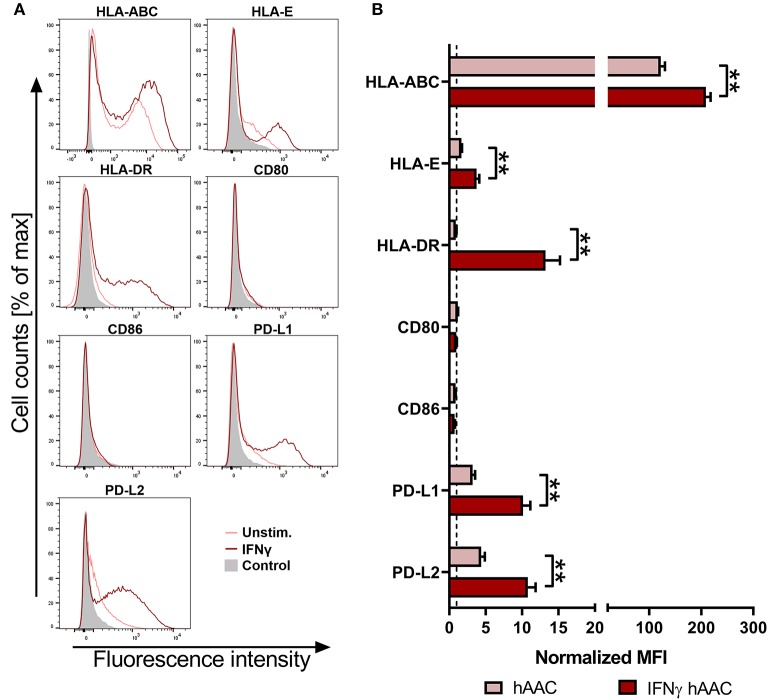
Immune phenotype of cryopreserved hAACs under constitutive and inflammatory conditions. **(A)** Representative histogram overlays for one hAAC donor display the expression pattern for immunologically-relevant surface markers (HLA-ABC, HLA-E, HLA-DR, CD80, CD86, PD-L1, PD-L2). Cells were cultured for 48 h without additional stimulation (hAAC; light red line) or in presence of 100 ng/mL human interferon-gamma (IFNγ hAAC; dark red line). After harvest by application of trypsin, cells were stained with human-specific antibodies and analyzed by flow cytometry. Fluorescence intensity of marker expression is presented compared to the unlabeled control (Control; filled gray curve). **(B)** Summarized hAAC surface marker expression data are presented as normalized mean of fluorescence intensities (MFI), that are calculated based on the respective controls (set to one; dashed black line), and shown as mean + SEM (*n* = 6; three independent experiments with six different hAAC donors). Differences between unstimulated hAACs and IFNγ hAACs were considered significant when ^**^p ≤ 0.01 with the Mann-Whitney *t*-test.

To investigate the surface marker profile in an inflammatory milieu, that mimics the environmental site of cardiac injury, hAACs were stimulated with 100 ng/mL of IFNγ for 48 h. Initial experiments with stimulation by pro-inflammatory cytokines (IFNγ or a combination with TNFα) for 1, 2, and 5 days, showed an increase in surface marker expression in the majority of tested markers (HLA-ABC, HLA-DR, PD-L1) with an overall maximum of up-regulation after 2 days (Supplementary Figure 2). Hence, the time point of 48-h stimulation with an appropriate IFNγ concentration of 100 ng/mL was chosen to determine the relative expression levels as normalized mean fluorescence intensities (MFI) for a set of immunologically-relevant markers. Stimulation of hAACs induced similar changes in the surface marker expression of all six donors as shown in FACS histogram overlays (Figure 2A) of one representative donor and summarized as normalized MFI values (Figure 2B) as well as percentages of marker positive cells (Supplementary Figure 3). All donors expressed HLA-class I (HLA-ABC, and partly HLA-E) and low or negligible levels of HLA class II (HLA-DR) constitutively, but significantly up-regulated both HLA-molecule classes under IFNγ-stimulation. In contrast, co-stimulatory molecules such as CD80 and CD86 were completely absent, even under stimulation. Additionally, a significant increase of the MFI for the immunomodulatory PD-1 ligands (PD-L1 and PD-L2) could be determined after stimulation with IFNγ (Figure 2B). Both markers were shown to be expressed on a considerable proportion of cells (Supplementary Figure 3). However, stimulation with IFNγ did not led to alterations in MFI or frequency of mesenchymal marker expression (CD29, CD44, CD73) on hAACs and even CD90 remained unchanged (Supplementary Figure 4).

### hAACs Evade Recognition by Allogeneic T Cells *in vitro* Even in an Inflammatory Milieu

**Figure 3:**
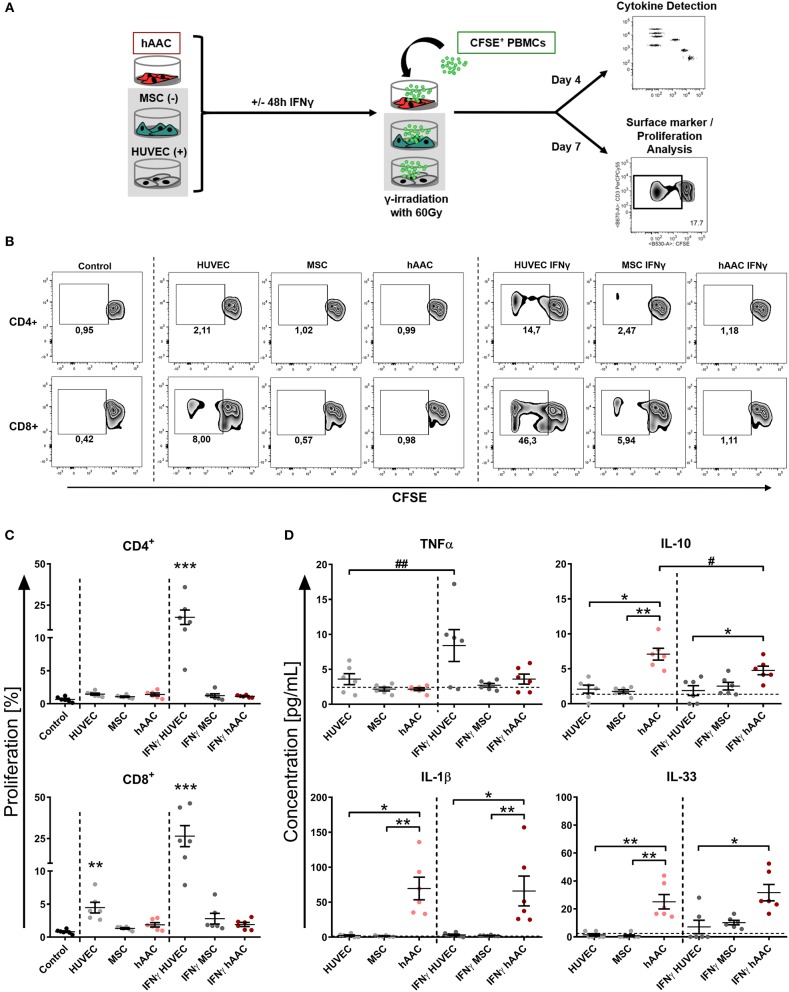
hAACs maintain a low-immunogeneic profile even in an inflammatory environment. **(A)** The experimental setup for analyzing hAACs immunogenicity in immune cell co-cultures is illustrated: mesenchymal stromal cells from the umbilical cord (MSCs) as well as human umbilical vein endothelial cells (HUVECs) served, respectively, as cellular controls [MSC (-); HUVEC (+)] and were cultured along with hAACs for 48 h in the presence or absence of 100 ng/mL IFNγ. The cells were gamma-irradiated with 60 Gray before carboxyfluorescein succinimidyl ester (CFSE)-labeled, human leukocyte antigen (HLA)-mismatched peripheral blood mononuclear cells (PBMCs) were either added to the adherent cell cultures of all three cell types or left alone as control. After 4 days of incubation, supernatants were taken for cytokine detection using the Legendplex™ human inflammation panel and after 7 days PBMCs were harvested, stained with human immune cell specific antibodies and analyzed flow cytometrically. Levels of CD4^+^ and CD8^+^ T cell proliferation were detected by determining reduced CFSE signal intensity (black square). **(B)** Representative flow cytometry plots of proliferated CD4^+^ and CD8^+^ T cells are shown for all PBMC co-culture groups and PBMCs only (Control). **(C)** Summarized proliferation data for CD4^+^ and CD8^+^ T cells are presented as mean ± SEM (*n* = 6; three independent experiments with six different hAAC donor). Groups were considered significantly different compared to the Control when ^*^p ≤ 0.0*5;* ^**^p ≤ 0.0*1;* ^***^p ≤ 0.001 with Kruskal Wallis ANOVA and Dunn's post-test. **(D)** Measured cytokine levels in [pg/mL] for TNFα, IL-10, IL-1β, and IL-33 are shown as mean ± SEM (*n* = 6). Cytokine levels of the PBMC control groups are depicted as dotted gray line. Groups were considered significantly different when ^*^p ≤ *0.05;* ^**^p ≤ *0.01;* ^***^p ≤ *0.001* with Kruskal Wallis ANOVA and Dunn's post-test. Differences between treatments were considered significant when *#p* ≤ 0.0*5; ##p* ≤ 0.0*1* with ordinary two-way ANOVA and Sidak's post-test.

Next, we were interested in the response of T cells against allogeneic hAACs due to their key role as mediators of allo-recognition and rejection in adaptive immune responses. Accordingly, we mimicked the *in vivo* situation by co-culturing HLA-mismatched PBMCs with hAACs from six different donors and investigated the induction of allogeneic T cell responses by monitoring their activation as well as proliferation. The T cell immune responses induced by MSCs and HUVECs have been well-described in the literature and therefore both cell types were used as controls for absent or induced responses, respectively. As shown in the experimental setup (Figure 3A) hAACs as well as MSCs and HUVECs were cultured with or without pre-stimulation by IFNγ for 48 h, thereupon CFSE-labeled PBMCs from healthy donors were added to the cultures. After 7 days of co-culture, the surface marker expression and proliferation of T cells were analyzed by flow cytometry. Compared to the unstimulated PBMC control cultures, the presence of unstimulated HUVECs significantly induced proliferation of CD8^+^ T cells but had no effect on the CD4^+^ T cell compartment (Figures 3B,C). However, pre-stimulation with IFNγ led to highly elicited levels of both CD4^+^ and CD8^+^ T cell proliferation. A similar trend was observed in the activation status that was revealed by significantly increased percentages of HLA-DR^+^ T cells in the CD4^+^ and CD8^+^ subsets after IFNγ pre-stimulation (Supplementary Figure 5). hAACs on the other hand displayed low-immunogeneic properties analogous to the moderate levels of induced T cell proliferation detected in MSC co-cultures. Both, hAACs and MSCs did not induce significant changes in CD4^+^ or CD8^+^ T cell proliferation after IFNγ pre-stimulation as well as under unstimulated conditions (Figures 3B,C), neither did both cell types lead to increased expression levels of the activation marker HLA-DR on T cells (Supplementary Figure 5).

Additionally, supernatants were taken from co-cultures after 4 days of incubation and were evaluated for their content of various cytokines. Summarized data for TNFα, IL-10, IL-1β, and IL-33 are presented in Figure 3D. The amount of the pro-inflammatory cytokine TNFα significantly increased in IFNγ-stimulated HUVEC co-cultures. Contrarily, unstimulated hAAC co-cultures exclusively showed elevated levels of IL-10 release, that significantly decreased with IFNγ pre-stimulation. Significant increases of IL-1β concentrations were detected in co-cultures of unstimulated and IFNγ-stimulated hAACs. While the concentration of IL-33 also increased in unstimulated hAAC cultures, IFNγ-triggered co-cultures only showed a significant elevation compared to the cytokine levels secreted by HUVECs. Contrary to TNFα and IL-10, mono-cultures of hAACs already constitutively produced the cytokines IL-1β and IL-33 (Supplementary Figure 6). Other cytokines measured, like MCP-1 and IL-8, were also produced at a basal level by the adherent cells and showed no inordinate changes in PBMC co-cultures (Supplementary Figure 7). IFNα, IFNγ, IL-12p70, IL-17A, IL-18, and IL-23 were not detectable or only at negligible levels (data not shown).

### Immunomodulatory Efficiency of hAACs Is Considerably Enhanced After IFNγ Treatment

Activation of T cells and the resulting inflammatory responses after cardiac injury largely contribute to adverse remodeling and development of chronic heart diseases in patients. We therefore examined the ability of hAACs, either unstimulated or IFNγ pre-stimulated, to modulate an already ongoing T cell response in co-cultures with αCD3/αCD28 activated PBMCs. In parallel PBMC co-cultures with both control cell types (HUVECs and MSCs) were performed. After 72 h of co-culture, proliferation rates of CD4^+^ and CD8^+^ T cells and cytokine release were analyzed.

**Figure 4:**
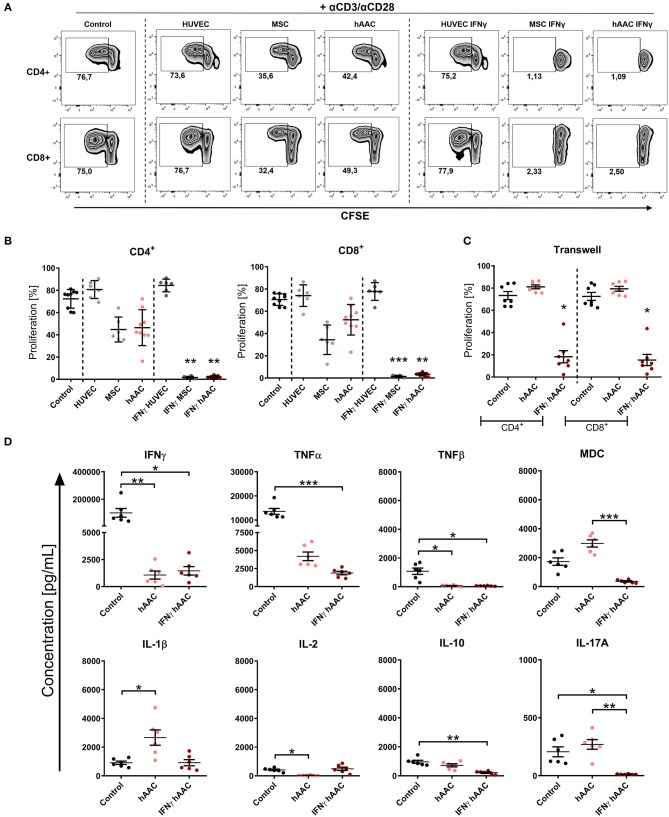
IFNγ pre-stimulation enhances the immune-modulatory capacity of hAACs. CFSE-labeled PBMCs were activated with anti-CD3/anti-CD28 antibodies (+αCD3/αCD28) and cultured alone (Control) or in the presence of unstimulated or IFNγ-stimulated hAACs, HUVECs or MSCs for 72 h. Cells were harvested, stained with human-specific antibodies and analyzed by flow cytometry for T cell proliferation, based on reduced CFSE signal intensity. Percentages of CD4^+^ or CD8^+^ proliferated cells for all experimental groups are shown as representative dot plots **(A)** and as summarized data with mean ± SEM **(B)** (*n* = 6–9; four independent experiments with seven different hAAC donors). **(C)** Experimental settings were repeated with hAACs under transwell culture conditions to evaluate a contact-dependent mode of action in the observed immune-modulatory effects (*n* = 7; three independent experiments with six different hAAC donors). **(D)** Supernatants of the direct immune cell co-cultures with either unstimulated or IFNγ-treated hAACs were analyzed with a Luminex bead kit for their content of IFNγ, TNFα, TNFβ, MDC, IL-1β, IL-2, IL-10, and IL-17A. Summarized data for cytokine concentrations [pg/mL] are presented as mean ± SEM (*n* = 6; three independent experiments with six different hAAC donors). Groups were considered significantly different when ^*^p ≤ *0.05;* ^**^p ≤ *0.01;* ^***^p ≤ *0.001* with Kruskal Wallis ANOVA and Dunn's post-test.

As expected, the presence of HUVECs did not significantly affect the proliferation rates of T cells (Figures 4A,B). Contrarily, hAACs and MSCs efficiently reduced the percentage of proliferating CD4^+^ and CD8^+^ cells, exclusively after IFNγ triggering, below 10% (Figures 4A,B). A slight reduction in T cell proliferation was observed with unstimulated adherent cells, but these changes were in fact not significant. Experimental settings were repeated with hAACs under transwell conditions to test for contact-dependency of the observed immunomodulatory effects. No changes in proliferation of CD4^+^ and CD8^+^ T cells were detectable with unstimulated hAACs in the transwell setting. Pre-stimulation with IFNγ on the other hand caused significant reduction of proliferation levels in both T cell subsets (Figure 4C). The same trend was observed with a significant decrease of the activation marker CD25 on CD4^+^ and particularly CD8^+^ T cells after IFNγ pre-stimulation (Supplementary Figure 8).

Furthermore, a significant decrease of IFNγ and TNFβ concentration was measured in co-cultures with unstimulated and IFNγ-stimulated hAACs, but only the latter showed a significant reduction in the amount of released TNFα, MDC, IL-10, and IL-17A. Interestingly, co-cultures with unstimulated hAACs produced significantly more IL-1β and less IL-2 (Figure 4D). Other cytokines measured like IL-5 and IL-13 showed significantly lower levels in IFNγ stimulated co-cultures (data not shown).

### Stimulation by IFNγ Leads to Specific Changes in the Gene Expression Profile of hAACs

**Figure 5:**
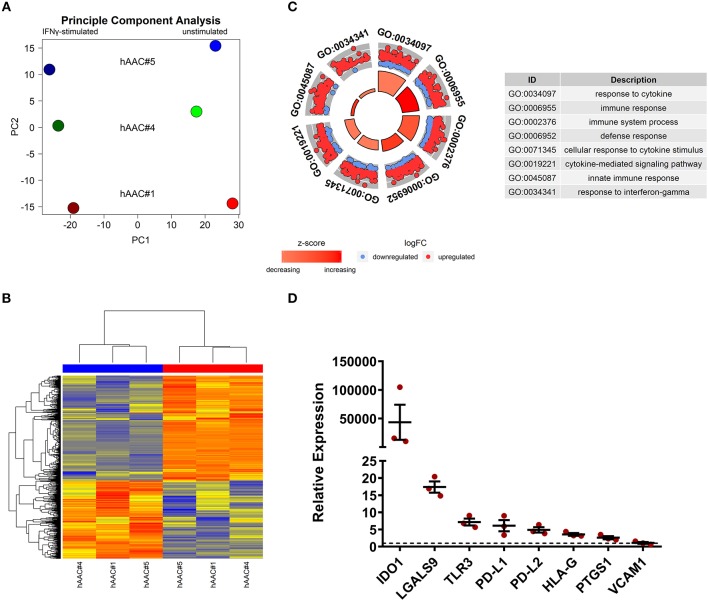
Whole genome expression analysis and quantitative verification of immunomodulatory gene expression of unstimulated and IFNγ pre-stimulated hAACs. **(A)** Microarray expression data of unstimulated and IFNγ-stimulated hAACs were normalized and log2-transformed. Thousand probe sets with the highest variances across all samples were selected and used in a principle component analysis, showing PC1 vs. PC2 for *n* = 3 hAAC donors (#1, #4, #5). **(B)** Differentially expressed probe sets were determined by fitting linear models to the data and apply Bayesian statistics. *P*-values were adjusted for multiple testing using false discovery rate. Probe sets with an adjusted *p*-value below.05 and a minimal absolute log2-foldchange of 1 are shown in the heatmap. The blue column bar indicates the unstimulated and the red bar the IFNγ pre-stimulated hAACs of donors #1, #4, #5. **(C)** Differentially expressed probe sets were used in an overrepresentation analysis utilizing the gene ontology system. The eight top-ranking results of the category biological process are shown. **(D)** The differentially expressed immunomodulatory genes *IDO1, LGALS9, TLR3, PD-L1, PD-L2, HLA-G, PTGS1* and *VCAM1*, identified in the global microarray analysis, were validated by qPCR. Values of IFNγ pre-stimulated hAACs were normalized to the unstimulated samples (set to 1; black dotted line) by means of a ΔΔCt analysis and upregulation is shown as mean of relative expression ± SEM for *n* = 3 hAAC donors (#1, #4, #5).

Whole genome gene expression of unstimulated and IFNγ pre-stimulated hAACs were analyzed on human hgu133plus2 microarrays (Affymetrix). Data were normalized and the 1000 most variable probe sets were used in a principle component analysis. This unbiased analysis revealed a strong separation of unstimulated and IFNγ pre-stimulated samples along the first principle component reflecting the experimental design. In addition, samples were separated according to different hAAC donors in the principle component two, indicating some heterogeneity of gene expression (Figure 5A). Although showing these individual characteristics in gene expression, additional analyses with Raman spectroscopy revealed a similar global phenotype of the hAAC cell product with no detectable differences in the molecular composition of cells derived from three hAAC donors (Supplementary Figure 9). Next, differentially expressed genes between the unstimulated and IFNγ pre-stimulated groups were determined by fitting linear models to the data and running a Bayesian statistic. Despite the genetic heterogeneity of the donors, a common response to IFNγ can be identified as shown in the heatmap (Figure 5B). Differentially expressed genes were subjected to an overrepresentation analysis utilizing the gene ontology system. The eight top-ranking results of the category “biological process” are shown in Figure 5C and were related to the immune system, cytokine-signaling and according to the experimental setup to the IFNγ-response. Remarkably, much more genes were up-regulated as down-regulated in the gene sets matched to these GO-terms. The whole results of the overrepresentation analysis are summarized in Supplementary Table 3. To identify a common mode of action among the hAAC donors after IFNγ pre-stimulation, the results of the genome-wide gene expression profile were checked for differential expression of known immunomodulatory genes in MSCs, including: *IDO1, PD-L1, PD-L2, NT5E, TGFB1, PTGES2, HGF, IGF, TNFAIP6, JAG1, ICOSLG, HLA-G, PGE2, IL-10, LDHB, LDHA, LGALS1, LGALS9, TLR3, ANXA1, VCAM1, PTGS1, PTGS2* and *LIF*. However, only *IDO1, LGALS9, TLR3, PD-L1, PD-L2, PTGS1, HLA-G*, and *VCAM1* were differentially expressed among all three donors and were therefore validated by qPCR. The analysis of the relative gene expression normalized to the corresponding unstimulated hAAC sample showed a very strong upregulation of *IDO1* expression in IFNγ-triggered hAACs. Yet, other immune regulatory genes like *LGALS9* (Galectin-9), *TLR3, HLA-G, PTGS1* (COX-1) as well as *PD-L1* and *PD-L2* also showed a distinct upregulation in the presence of IFNγ, but admittedly to a much lesser extent (Figure 5D).

### IDO Predominantly Mediates Immunomodulation by IFNγ-Triggered hAACs and Acts Through Apoptosis of T Cells

**Figure 6:**
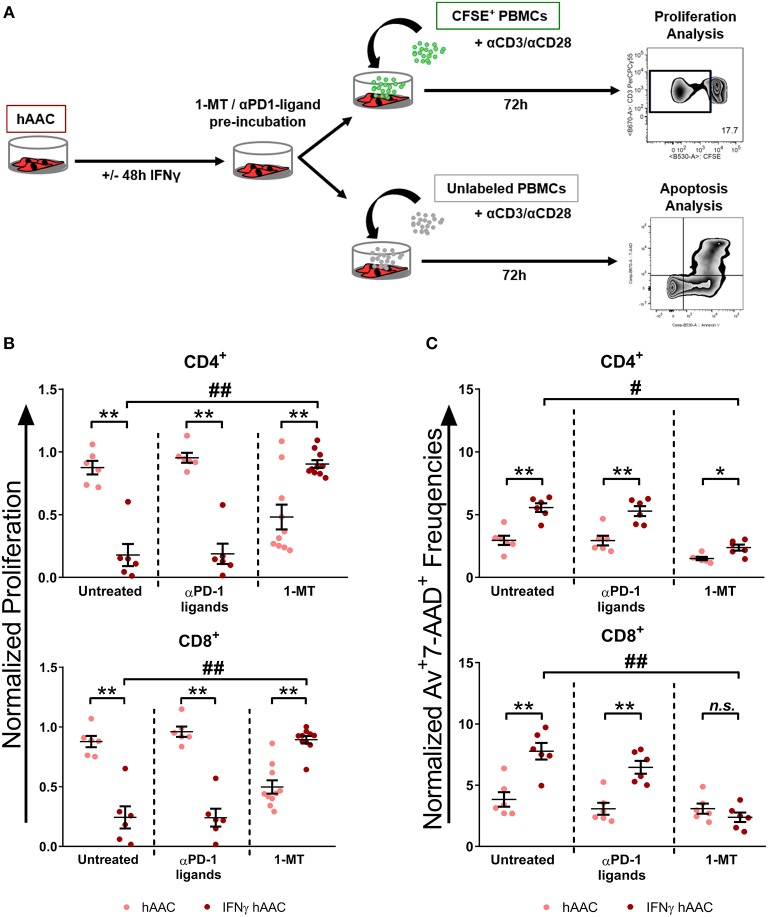
Inhibition of T cell proliferation by hAACs is indoleamine-2,3-dioxygenase (IDO)-dependent and involves apoptosis. **(A)** The experimental setup to evaluate the potential involvement of IDO and programmed death-1 (PD-1) ligands in hAAC effects on T cells is shown schematically: hAACs were seeded and stimulated with IFNγ or left unstimulated for 48 h. Before co-cultivation with immune cells, 1-mehtyl-L-tryptophan (1-MT) as specific inhibitor of IDO or blocking antibodies against PD-L1 and PD-L2 (αPD1 ligands) were applied. Subsequently, either CFSE-labeled or unlabeled PBMCs were activated by adding a cocktail of anti-CD3/anti-CD28 (+αCD3/αCD28) antibodies and left alone as controls or cultured with hAACs for 72 h. CD4^+^ or CD8^+^ T cell proliferation levels as well as the percentage of Annexin-V^+^ (Av+) 7-AAD^+^ late apoptotic cells were determined by flow cytometry. **(B)** The normalized proliferation values for CD4^+^ or CD8^+^ T cell subsets were calculated based on the respective PBMC controls and are presented as mean ± SEM (*n* = 6–10; four independent experiments with seven different hAAC donors). **(C)** The normalized percentages of late-apoptotic Annexin-V^+^ (Av^+^) 7-AAD^+^ cells within the CD4^+^ and CD8^+^ T cell subsets were calculated based on the respective PBMC controls and are presented as mean ± SEM (n = 6; two independent experiments with six different hAAC donors). Unstimulated (hAAC) and IFNγ-stimulated hAACs (IFNγ hAAC) within the same treatment were considered significantly different when ^*^p ≤ *0.05;* ^**^p ≤ *0.01* with the Mann-Whitney *t*-test. Differences between treatments were considered significant when *#p* ≤ *0.05; ##p* ≤ *0.01* with Kruskal Wallis ANOVA and Dunn's post-test.

Since *IDO1* was by far the most upregulated gene in IFNγ pre-stimulated hAACs (Figure 5D) and PD-L1/PD-L2 were already described in the literature as a key mechanism of action in cardiac-derived cells (30) and MSCs (45), we selected them as promising molecules that might be involved in the observed immunomodulatory potential of hAACs (Figure 4B). As illustrated in Figure 6A, hAACs were cultured for 48 h in the presence or absence of IFNγ. Following 2 h of pre-incubation with 1-MT as a specific inhibitor of IDO or 12 h with blocking antibodies against PD-L1 and PD-L2 (αPD1-ligand), either CFSE labeled or unlabeled PBMCs were added to the cultures and were subsequently activated with a cocktail of αCD3/αCD28 antibodies. After 72 h, PBMCs were analyzed by flow cytometry to determine proliferation and apoptosis of T cells.

hAACs without treatment by blocking agents confirmed the observed immunomodulatory effects by significantly inhibiting T cell proliferation after IFNγ pre-stimulation, shown as relative proliferation normalized to the αCD3/αCD28 PBMC control (Figure 6B). Additionally, significantly higher levels of late-apoptotic T cells occurred in IFNγ pre-stimulated co-cultures, shown as relative percentages of Annexin-V^+^7-AAD^+^ T cells normalized to the αCD3/αCD28 PBMC control (Figure 6C). Treatment with blocking antibodies against both PD-1 ligands neither had a significant effect on proliferation (Figure 6B), nor on apoptosis rates (Figure 6C) of CD4^+^ and CD8^+^ T cells when compared to the untreated control group. Notably, treatment with 1-MT, mediating the blocking of IDO, resulted in significant restoration of T cell proliferation in IFNγ-stimulated hAAC co-cultures. However, unstimulated cultures displayed rather reduced proliferation rates for both T cell subsets (Figure 6B). Complementary to the proliferation, treatment with 1-MT also caused significant reduction of Annexin-V^+^7-AAD^+^ late-apoptotic T cells for the IFNγ hAAC group relative to the untreated control group (Figure 6C). Yet, comparing unstimulated hAACs with IFNγ hAAC under 1-MT treatment illustrates, that levels were still elevated for apoptotic CD4^+^ cells, but were in fact not significantly different for the CD8^+^ subset (Figure 6C).

## Discussion

Based on the limited treatment options for cardiovascular diseases, the development of new and potent cellular therapeutics has emerged in the past decade as a promising new strategy aimed at preventing or even reversing myocardial damage.

A huge variety of cell therapy studies had been conducted with MSCs of different tissue origins, which reported only modest or no significant efficacy to improve cardiac function (46). The initial enthusiasm faded and a multitude of scientific questions remained unanswered (47). Apart from developing solutions for patient selection, timing of administration and appropriate application routes including scaffold-based approaches, efficient cell pre-conditioning or genetic manipulation (24), the most pressing task is the identification of an isolation procedure for a suitable and effective cell type. In this context, cells isolated from close origin to the target tissue seem to be the most promising cell therapeutic strategy (48, 49). Therefore, cardiac-derived cell types excelled as a potentially powerful cell source due to their known cardio-protective and pro-angiogenic effects. Due to the lack of scientific evidence and understanding regarding these positive effects of cell-based therapies, a return to the bench-side would not only help to understand the mode of action, but also may lead to a more reliable and effective therapy for the patient (12). Moreover, large batches of healthy and instantly available cells are needed for the realization of a broad and less expensive clinical application, which clearly favors allogeneic cell sources. To exclude any unwanted immunogenicity, especially under an inflammatory and disease-related condition, it is indispensable to analyze the interaction with immune cells *in vitro*. In the present study, we addressed these issues by analyzing first the main immunological characteristics of the allogeneic hAAC cell product to estimate its immunogenicity and secondly by investigating the immunomodulation capacity focusing on T cell responses.

We could convincingly show that human allogeneic cells from the right atrial appendage (hAACs) exhibit, even in an inflammatory environment, an inherently low immunogenicity, as demonstrated by the absence of adversely induced T cell responses in immune cell co-cultures. Moreover, we could clearly present that hAACs are effective modulators of already induced immune cell responses by suppressing T cell proliferation and pro-inflammatory cytokine release, especially after triggering with IFNγ. We could additionally show that IDO is one of the key mediators responsible for the observed immunomodulating features of hAACs. Our data strongly support the use of these allogeneic cardiac-derived cells for the therapeutic application in cardiovascular diseases.

So far, application of allogeneic cells for therapeutic approaches was only taken into account for mesenchymal cell types, like mesenchymal stromal cells (MSC) and cardiac progenitor cells (CPC) due to their known low immunogeneic phenotype (22, 25, 50, 51). Therefore, we checked our newly described hAAC cell product for the presence of characteristic mesenchymal cell markers and could confirm a similar expression pattern as seen in MSCs, with the only exception of a significantly reduced CD90 expression. A low CD90 expression might be an advantage for future therapeutic approaches by reducing fibrosis, as shown in a prospective analysis of a clinical trial using cardiosphere-derived cells (33). Inversely, another study using human tissue revealed that CD90 expressing fibroblasts are mainly responsible for the fibrotic thickening in peritoneal dialysis patients (52).

However, more important for the clinical application of an allogeneic cell product is to determine its potential immunogenicity (53, 54) by evaluating the expression of immunological markers under constitutive and inflammatory conditions. In the present study, we could clearly demonstrate that hAACs constitutively expressed HLA-ABC and partially HLA-E, but not HLA-DR as well as the main co-stimulatory molecules CD80 and CD86. After IFNγ stimulation, the expression profile shifted toward a further increased expression level of HLA-class I molecules and *de novo* induction of HLA-DR expression. Yet, the co-stimulation molecules remained absent. Additionally, hAACs showed expression of the immunomodulatory molecules PD-L1 and PD-L2 on a considerable proportion of cells that was further enhanced after inflammatory stimulation. Comparatively, this surface marker profile was already described for MSCs (45, 55, 56) and related cardiac cell types (30, 57).

Despite the upregulated expression of both classes of HLA-molecules after IFNγ stimulation, hAACs evaded an adaptive immune response and were thereby not able to trigger substantial CD4^+^ and CD8^+^ T cell proliferation, when co-cultured with human PBMCs. The in parallel tested human MSCs induced a similar proliferation response pattern in T cells. Although these results might suggest the conclusion that hAACs and MSCs share a common mode of action, they clearly differed in the spectrum of secreted cytokines within PBMC co-culture supernatants. Whereas, unstimulated and IFNγ stimulated MSCs and hAACs did not induce any TNFα release, only the cardiac mesenchymal-like hAACs showed significantly enhanced induction of the anti-inflammatory cytokine IL-10 in both conditions. However, it is striking, that exclusively IL-1ß and IL-33 levels were significantly higher in co-cultures with hAACs, which exceeded the constitutive secretion of both cytokines by hAACs alone. So far it is unclear, which effects both cytokines of the IL-1 family would have after transplantation of hAACs in a therapeutic setting. It is known that they are classical pro-inflammatory factors with an ambivalent function. On the one hand, IL-1β secretion of MSCs is involved in monocyte dependent regulation of CD4^+^ and CD8^+^ T cell activation by triggering the release of TGFβ (58). IL-33, on the other hand, could play an important role in cardiac tissue preservation and repair in response to myocardial injury (59–62).

Our results regarding the induction of immune cell responses with CD90^low^ hACCs are comparable to data described for CD90^+^ allogeneic human CPCs (30). Therefore, the expression or absence of CD90 on cardiac-derived cells apparently seems to be of inferior importance for the immunogenicity of the respective allogeneic cell product. However, in contrast to our study Lauden et al. demonstrated a rather low, but significant induction of CD4^+^ T cell proliferation. Consequently, they were able to prove the induction of regulatory T cells in co-cultures of purified CD4^+^ T cells with human CPCs.

For the potential clinical application of the hAAC cell product, not only a low immunogenicity, but also the capacity to suppress ongoing inflammatory immune responses is important, since it is widely believed to be responsible for the adverse remodeling after cardiac injury (63, 64). In co-cultures with human allogeneic PBMCs, we were able to demonstrate the capacity of hAACs as well as MSC controls to suppress CD4^+^ and CD8^+^ T cell proliferation nearly to the same extent. Both cell types inhibited T cell proliferation by trend if cells were cultured under unstimulated conditions and strongly enhanced their suppressive capacity after IFNγ pre-stimulation. The “licensing” effect of IFNγ was already well-described by several groups for MSCs from different sources (65–69). In contrast, no clear similarities between human CPCs and MSCs were detectable regarding the inhibitory capacity of T cell proliferation in PHA stimulated immune cell cultures as well as in mixed lymphocyte reaction settings (30). However, the experimental design of our study relied on different cell sources and a diverging setup of immune cell co-cultures, which might explain varying results of the immunomodulation. The comparable immunomodulatory efficacy of CD90^low^ hAACs and the CD90^+^ control umbilical cord MSCs argues, that CD90 expression does not play a fundamental role in our experimental setup. Although, a potential correlation was described by others for human MSCs derived from bone marrow, amnion and chorion (70), other factors seem to determine the immunosuppressive features of this specific hAAC product.

The high potency of hAACs to efficiently down-regulate already ongoing immune responses was additionally confirmed by a strong suppression of the pro-inflammatory mediators IFNγ, TNFα, and IL-17A as well as IL-2 to a minor extent. Again, these effects were further enhanced in co-cultures with IFNγ-licensed cells. The typical induction of a shift from an inflammatory toward a more anti-inflammatory secretion profile was described for human MSCs (71–74) and for CPCs (25, 75). However, presence of hAACs in triggered immune cell co-cultures rather lowered anti-inflammatory cytokines like TGFß and IL-10 instead of inducing those as described for MSCs (58, 73, 76).

Next, we wanted to get a deeper understanding of the molecular changes in hAACs after pre-stimulation with IFNγ that are likely to be responsible for the immunosuppressive or modulatory efficacy. For this, we compared unstimulated and IFNγ stimulated hAACs in a whole genome analysis by Affymetrix® microarray technology and could determine similar characteristics for all three donors between each treatment group, despite visible biological heterogeneities in their individual RNA profiles. More interestingly, the global analysis revealed, that pathways of general immune system responses (innate and adaptive), as well as cytokine signaling and IFNγ responses were preferentially involved. A closer look into specific, immune-relevant molecules, that were significantly up-regulated under IFNγ stimulation, exposed *IDO1* as one of the strongest expressed genes among other interesting candidates, such as *LGALS9* (Galectin-9), *TLR3, HLA-G, PTGS1* (COX-1). The particularly high degree of *IDO1*-upregulation in IFNγ pre-stimulated hAACs was also confirmed by qPCR. IDO is often discussed to be involved in immunosuppressive effects exerted by MSCs on T cells by depletion of tryptophan and accumulation of metabolites like kynurenin (77–81). In correlation with the strong up-regulation of IDO, we could prove the involvement of this particular molecule in the suppression of T cell proliferation and the induction of their apoptosis by application of the specific inhibitor 1-MT. These observations are in accordance with a recent study, which correlated the suppressive capacity of IFNγ licensed MSCs on third party T cell proliferation to enhanced IDO and PD-L1 expression (82).

Interestingly, PD-L1 and PD-L2 were found to be up-regulated on RNA and protein level in hAACs after inflammatory stimulation. The interaction of these two molecules with PD-1 on T cells might also contribute to the observed immunosuppressive effects. For bone marrow MSCs it was recently demonstrated that the expression and secretion of both PD-1 ligands mediated suppression of CD4^+^ T cell activation, down-regulated IL-2 secretion and induced hypo-responsiveness and cell death in T cells (45). However, in our experimental settings the application of blocking antibodies against both PD-1 ligands could neither reverse the suppression of T cell proliferation nor prevent the induction of apoptosis. We also found, that IFNγ pre-stimulated hAACs showed nearly the same suppression of αCD3/αCD28-induced T cell proliferation in direct contact as well as transwell settings. That implies that hAACs mainly mediate their immunomodulatory effects in an inflammatory milieu by soluble factors or vesicles that are able to cross the transwell membrane. A paracrine mode of action has often been suggested by others for MSCs and related mesenchymal cell types (83–86). The missing blocking effect of PD-1 ligands, which are secreted and also expressed on the cell surface, clearly discriminates hAACs from the before mentioned CD90^+^ CPCs, that exert a more contact-dependent mode of suppression by PD-L1 involvement on ongoing immune responses (30). Conclusively, the observed increase of PD-L1 and PD-L2 expression on the cell surface seems to play only an inferior role in the immune regulation mediated by hAACs. Even though their general cellular characteristics are clearly distinguishable from fibroblasts and conventional MSCs (32), hAACs behave more like MSCs in this immunological context. The importance of the observed up-regulation of other potential immunoregulatory genes such as Galcetin-9 for the immunomodulatory capacity of hAACs has to be analyzed in more detail in future studies.

There are also still open questions remaining regarding the nature of how hAACs avoid unwanted immune responses in clinically relevant settings of cardiac injury. Apart from the proved involvement of IDO in the efficient T cell immunosuppression under IFNγ treatment, the interaction of antigen-presenting cells, like monocytes, has also to be taken into account. In this regard, it became evident that MSCs could skew monocytes toward anti-inflammatory macrophages (87–89) and induce the generation of regulatory T cells (58, 76). Additionally, it was found that MSCs could be taken up by monocytic cells and induce changes toward a non-classical monocyte phenotype with enhanced expression of PD-L1 and secretion of IL-10 that subsequently modulates adaptive immune responses (90). Furthermore, it was recently published that human CPCs attracted monocytes by a set of released cytokines and mediators and consequently changed the polarization of differentiating macrophages toward an M2-type cell (25, 91). Future studies will therefore focus on this hAAC/antigen-presenting cell interplay to gain a better understanding of the underlying mechanism of action and ensure the safe and effective translation of this hAAC cell product.

**Figure 7:**
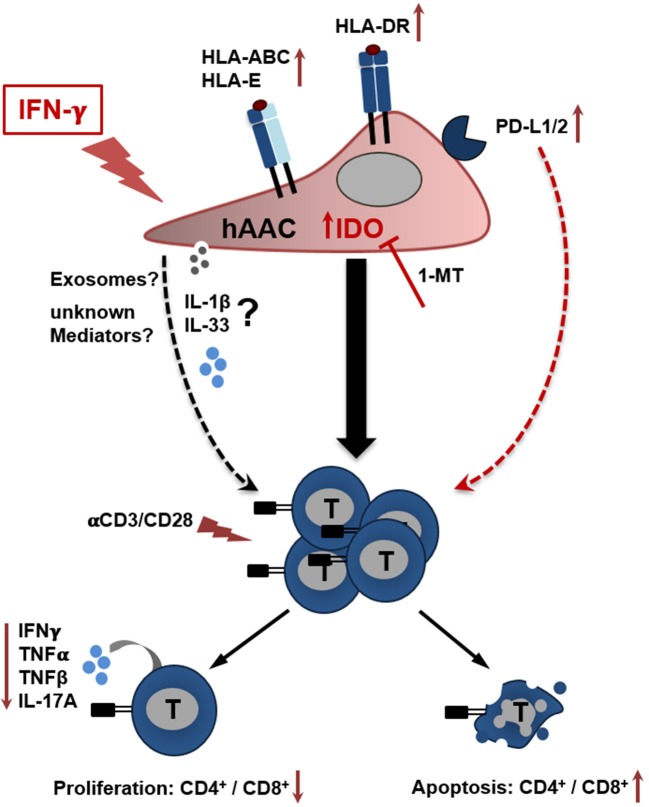
Potential crosstalk between hAACs and T cells in an inflammatory milieu. The scheme illustrates the impact of IFNγ stimulation on CD90^low^ hAACs and its implication on the interplay with αCD3/αCD28 activated human T cells. Stimulation of hAACs alone induces upregulation of HLA-class I (HLA-ABC, HLA-E) and *de novo* expression of HLA-class II molecules (HLA-DR). Even though expression of the inhibitory co-stimulatory molecules PD-L1 and PD-L2 is enhanced on the cell surface, no specific effects in the interaction with activated T cells were detectable. Similarly, the role of the specific secretion of IL-1 family cytokines (IL-1β, IL-33) by hAACs and additional unknown mediators like exosomes remain unclear. However, a global genome-wide expression profile analysis revealed IDO as one of the strongest expressed genes in IFNγ stimulated hAACs. Specific blocking by 1-MT proved the essential involvement of IDO in the interaction with T cells. It mediates the suppression of CD4^+^ and CD8^+^ T cell proliferation, reduces the secretion of pro-inflammatory cytokines (IFNγ, TNFα, TNFβ, IL-17A) and induces apoptosis of both T cell subsets. We hypothesize, that this mode of hAAC interaction might contribute to the resolution of immune responses and limit thereby the effects of adverse remodeling after cardiac injury.

In the present study we could show, that CD90^low^ hAACs isolated from human heart tissue represent a new allogeneic off-the-shelf mesenchymal-like cell product with therapeutically interesting features. Most importantly, hAACs do not trigger immunogeneic effects based on the low expression of HLA-DR and absence of co-stimulatory molecules after pro-inflammatory IFNγ stimulation. Moreover, hAACs clearly demonstrated a strong potential to inhibit ongoing immune responses even in inflammatory environments. Ultimately, we could illustrate that IDO-upregulation under IFNγ pre-treatment seems to be one of the most important players mediating the observed suppressive effects (Figure 7). However, the involvement of other so far not identified soluble factors cannot be excluded. This paracrine mode of action would also suggest the opportunity to isolate and use extracellular vesicles derived from hAACs for a clinical application. Our data in general would argue for a safe application of the hAAC cell product in an allogeneic setting, which also facilitates high potential to suppress already ongoing immune responses and thereby limit the progression of adverse remodeling after cardiac injury.

## Data Availability

The genome-wide Affymetrix® gene expression profile datasets generated for this study can be found in the Gene Expression Omnibus repository: https://www.ncbi.nlm.nih.gov/geo/query/acc.cgi?acc=GSE126461. The remaining raw data supporting the conclusions of this manuscript will be made available by the authors, without undue reservation, to any qualified researcher.

## Ethics Statement

Human atrial appendage-derived cells (hAACs) were isolated from atrial appendages of eight donors according to the local guidelines of the Charité-Universitätsmedizin Berlin as well as the Declaration of Helsinki and the study was approved by the ethics committee of the Charité-Universitätsmedizin Berlin (No. 4/028/12). Human peripheral blood mononuclear cells (PBMCs) were isolated from buffy coats (German Red Cross, Berlin, Germany; approved by the local Ethical Committee, EA1/226/14). Umbilical cord mesenchymal stromal cells were obtained for human cell and tissue sample collection from the Institutional Review Board of the Medical University of Graz (protocol 19-252 ex 07/08). Umbilical cord samples were collected from mothers that gave written informed consent after full-term pregnancies in accordance with the Declaration of Helsinki.

## Author Contributions

FD led the project and was responsible for study design, execution of experiments, collection and assembly of data, data analysis, and interpretation, as well as manuscript writing. MSt established methods, performed experiments, and interpreted data. KJ designed experimental approaches, performed and interpreted the microarray analyses and revised the manuscript. MH and MSi supplied the study materials, provided administrative support, and revised the manuscript. MSe was responsible for the project conception and design, administrative support, data analysis and interpretation, manuscript writing, and final approval of the manuscript.

### Conflict of Interest Statement

MSi and MH are inventors of patent family of EP2129774B1 (Cells for heart treatment). MSi is shareholder of CellServe GmbH (Berlin, Germany) and BioRetis GmbH (Berlin, Germany). CellServe GmbH holds a license of the above patent family. The remaining authors declare that the research was conducted in the absence of any commercial or financial relationships that could be construed as a potential conflict of interest.

## Footnotes

^1^European Cardiovascular Disease Statistics 2017 edition. *CVD Statistics 2017* (2017). Available online at: http://www.ehnheart.org/cvd-statistics/cvd-statistics-2017.html (accessed: February 02, 2019).

^2^R Core Team. *R: A Language and Environment for Statistical Computing*. Vienna: R Foundation for Statistical Computing (2018). Available online at: https://www.R-project.org/

## Abbreviations

1-MT: 1-methyl-L-tryptophan
CardAPs: cardiac-derived adherent proliferating cells
CD: cluster of differentiation
CFSE: carboxyfluorescein succinimidyl ester
COX-1: cyclooxygenase 1
CPCs: cardiac progenitor cells
hAACs: human atrial appendage-derived cells
HLA: human leukocyte antigen
HUVECs: human umbilical vein endothelial cells
IDO: indolamine-2,3-dioxygenase
IFNγ: interferon-gamma
IL: interleukin
MFI: mean fluorescence intensity
MSCs: mesenchymal stromal cells
PBMCs: peripheral blood mononuclear cells
PD-L: programmed death ligand
TGFβ: transforming growth factor beta
TLR3: toll-like receptor 3
TNF: tumor necrosis factor.
