# Supplementary material for: Enhanced Immunomodulation in Inflammatory Environments Favors Human Cardiac Mesenchymal Stromal-Like Cells for Allogeneic Cell Therapies
Source: Front Immunol. 2019 Jul 23;10:1716. doi: 10.3389/fimmu.2019.01716 (PMC6665953; doi:10.3389/fimmu.2019.01716)
Supplement: Supplementary file 1 [file Data_Sheet_1.docx]

Supplementary Material

# Supplementary Tables

**Supplementary Table 1.** HLA genotypes of different hAAC, MSC and PBMC donors.

hAACs, MSCs and PBMCs were genotyped for HLA-A, HLA-B and DRB1 to survey allogenicity in proliferation induction and immunomodulation experiments.

|  | HLA-A | | HLA-B | | DRB1 | |
| --- | --- | --- | --- | --- | --- | --- |
| hAAC1 | 02 | 23 | 40 | 49 | 11 | 13 |
| hAAC2 | 03 | 32 | 07 | 57 | 07 | 15 |
| hAAC3 | 03 | 11 | 15 | 50 | 07 | 09 |
| hAAC4 | 03 | - | 35 | 49 | 01 | 07 |
| hAAC5 | 01 | 03 | 27 | 57 | 07 | 15 |
| hAAC6 | 01 | 03 | 07 | 08 | 01 | 03 |
| hAAC7 | 02 | 26 | 18 | 37 | 11 | 13 |
| hAAC8 | 01 | 02 | 08 | 57 | 15 | 16 |
| MSC1 | 02 | 24 | 15 | 44 | 11 | 13 |
| PBMC1 | 01 | 31 | 38 | 51 | 09 | 13 |
| PBMC2 | 11 | - | 07 | 35 | 01 | 15 |
| PBMC3 | 11 | 30 | 13 | 15 | 04 | 07 |
| PBMC4 | 02 | 03 | 15 | 40 | 13 | 13 |
| PBMC5 | 24 | 68 | 40 | 51 | 01 | 03 |
| PBMC6 | 24 | 26 | 13 | 44 | 01 | 07 |
| PBMC7 | 01 | 02 | 18 | 57 | 03 | 13 |
| PBMC8 | 24 | - | 07 | 44 | 01 | 04 |

**Supplementary Table 2.** Overview of antibodies and dyes used for staining.

| Antibody/Dye name | Clone | Fluorochrome | Company | Dilution | Catalog# | RRID |
| --- | --- | --- | --- | --- | --- | --- |
| HLA-ABC | W6/32 | APCCy7 | Biolegend | 1:100 | 311425 | AB_10708421 |
| HLA-E | 3D12 | APC | Biolegend | 1:50 | 342605 | AB_2565260 |
| HLA-DR | L243 | PECy7 | Biolegend | 1:600 | 307616 | AB_493588 |
| CD3 | SK7 | PerCPCy5.5 | BD Biosciences | 1:20 | 332771 | - |
| CD4 | OKT4 | APC | Biolegend | 1:50 | 317416 | AB_571945 |
| CD8 | BW135/80 | PE | Miltenyi Biotec | 1:200 | 130-091-084 | AB_244338 |
| CD14 | MφP9 | APCCy7 | BD Biosciences | 1:100 | 557831 | AB_396889 |
| CD25 | BC96 | PerCPCy5.5 | Biolegend | 1:50 | 302626 | AB_2125478 |
| CD29 | TS2/16 | PE | Biolegend | 1:100 | 303004 | AB_314320 |
| CD31 | WM59 | FITC | BD Biosciences | 1:50 | 555445 | AB_395838 |
| CD34 | 4H11 | FITC | Biolegend | 1:50 | 316405 | - |
| CD44 | IM7 | PECy7 | Biolegend | 1:3000 | 103030 | AB_830787 |
| CD45 | HI30 | PacificBlue | Biolegend | 1:200 | 304022 | AB_493655 |
| CD45 | 2D1 | PerCP | BD Biosciences | 1:100 | 345809 | - |
| CD54 | HCD54 | FITC | Biolegend | 1:1000 | 322720 | AB_2121926 |
| CD73 | AD2 | APC | Biolegend | 1:1000 | 344005 | AB_1877158 |
| CD80 | 2D10 | FITC | Biolegend | 1:20 | 305206 | AB_314502 |
| CD86 | IT2.2 | PE | Biolegend | 1:50 | 305406 | AB_314526 |
| CD90 | 5E10 | PerCPCy5.5 | Biolegend | 1:400 | 328117 | AB_961312 |
| CD105 | 43A3 | PE | Biolegend | 1:50 | 323205 | AB_755957 |
| CD106 | STA | PE | Biolegend | 1:50 | 305806 | AB_314562 |
| c-Kit (CD117) | 104D2 | APC | Invitrogen | 1:50 | CD11705 | AB_2536476 |
| CD166 | 3A6 | PE | Biolegend | 1:50 | 343903 | AB_2289303 |
| CD166 | 3A6 | PerCPCy5.5 | BD Biosciences | 1:50 | 562131 | AB_10897166 |
| PD-L1 (CD274) | 29E.2A3 | PerCPCy5.5 | Biolegend | 1:100 | 329738 | AB_2617010 |
| PD-L1 (CD274) | 29E.2A3 | - | Biolegend | 5µg/mL | 329702 | AB_940372 |
| PD-L2 (CD273) | MIH18 | APC | Biolegend | 1:100 | 345508 | AB_2162176 |
| PD-L2 (CD273) | MIH18 | - | Biolegend | 5µg/mL | 345502 | AB_1953319 |
| Annexin-V | - | FITC | Biolegend | 1:25 | 640906 | AB_2561292 |
| CFSE | - | - | Biolegend | 5µM | 423801 | - |
| 7-AAD | - | 7-AAD | Biolegend | 1:25 | 420404 | - |
| Wheat Germ Agglutinin | - | CF488A | Biotium | 5µg/mL | 29022-1 | - |

**Supplementary Table 3.** Results of over-representation analysis of differential expressed genes in terms of the biological process branch of the gene ontology system. Probe sets were mapped to genes and functional annotations using the DAVID database.

(Please find the attached document “Table3.xlsx” for the complete Excel file with all the results.)

#
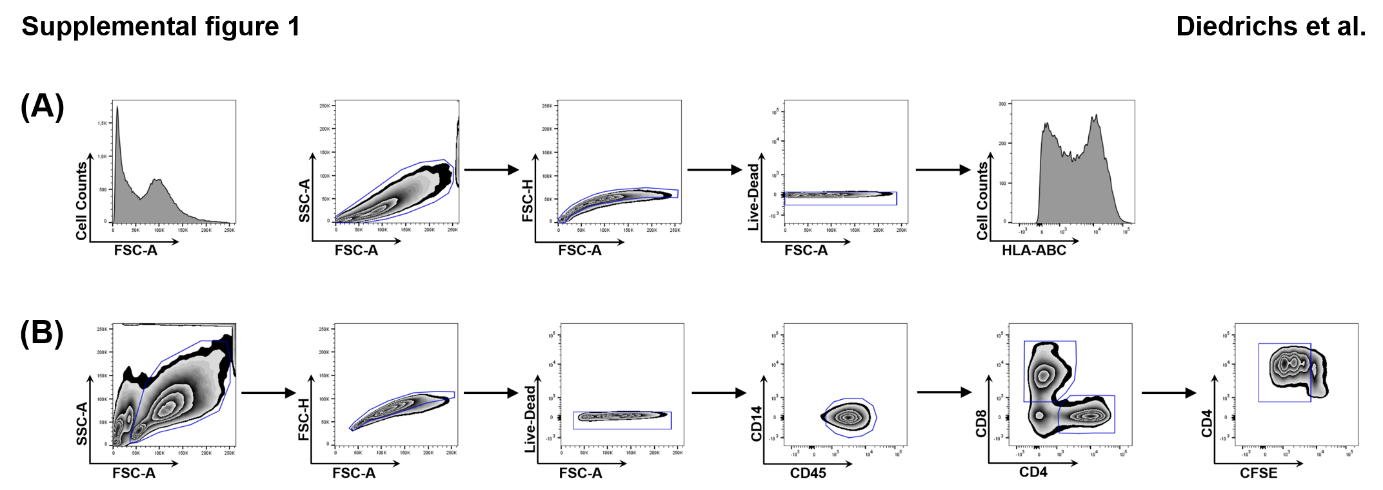
Supplementary Figures

**Supplementary Figure 1:** Flow cytometry gating strategies of mono-culture and co-culture experiments.

**(A)** The hAAC cell product features a heterogonous size distribution in the forward-scatter area (FSC-A) histogram. Therefore, both distinct size-subsets were included in the pre-gating on the FSC-A against sideward-scatter area (SSC-A) to analyze the complete cell product. Next, the resulting cells were gated on FSC-A against forward-scatter height (FSC-H) to discriminate between doublets and single cells within the population. To exclude apoptotic or dead cells and assure the viability of hAACs, cells were gated on FSC-A against Live-Dead marker. In a last step, fluorescence intensities of various markers were analyzed, exemplarily shown here for HLA-ABC. **(B)** In human PBMC cultures the leukocyte population was defined according to size and granularity (FSC-A vs. SSC-A). Doublets (FSC-A vs. FCS-H) and apoptotic or dead cells (FCS-A vs. Live-Dead marker) were excluded from analysis. Monocytes and any possible debris from the adherent cells were gated out (CD14 vs. CD45). In a last gating step T cells subsets were discriminated (CD4 vs. CD8), before proliferation as seen in reduction of CFSE signal intensity was determined for both subsets, exemplarily shown here for CFSE vs. CD4.


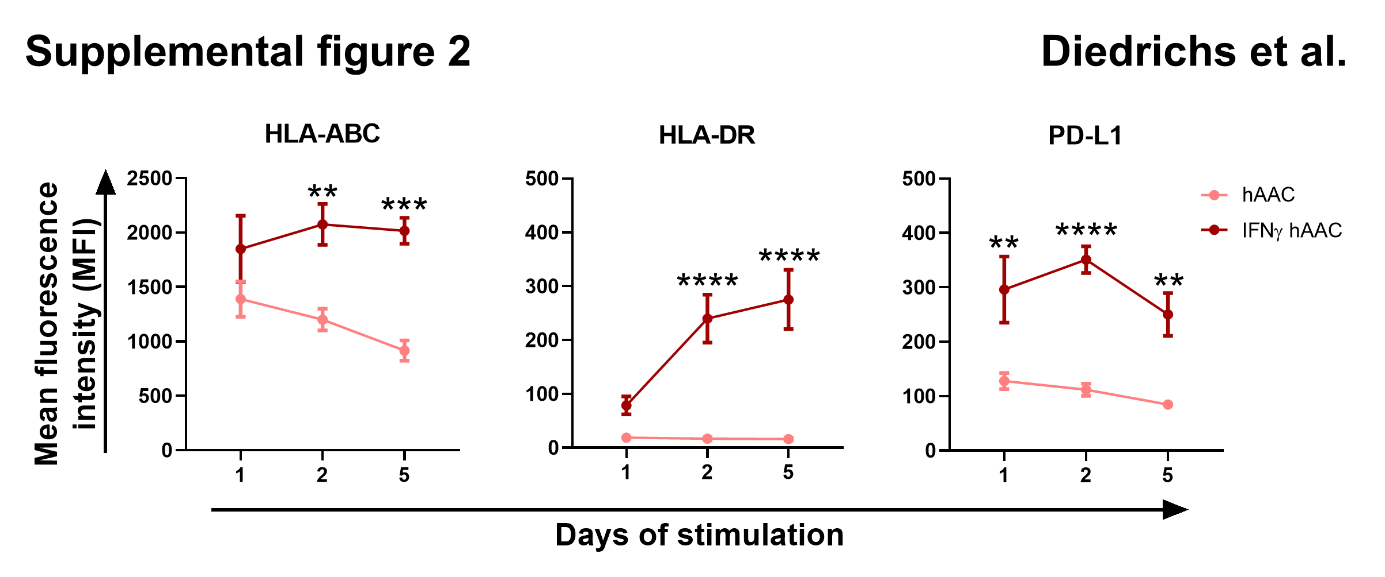
**Supplementary Figure 2:** Kinetic of immunological-relevant surface marker expression on hAACs.

Cells were cultured for one, two and five days without additional stimulation (hAAC; light red line) or in presence of 100 ng/mL human interferon-gamma (IFNγ hAAC; dark red line). After harvest by application of trypsin, cells were stained with human-specific antibodies against HLA-ABC, HLA-DR and PD-L1 and were analyzed by flow cytometry. Fluorescence intensity of marker expression is presented as mean ± SEM for all time points (n = 6; three independent experiments with six different hAAC donor). Differences between stimulations were considered significant when ***p* ≤ *.01; ***p* ≤ *.001; ****p* ≤ *.0001* with ordinary two-way ANOVA and Sidak’s post-test.


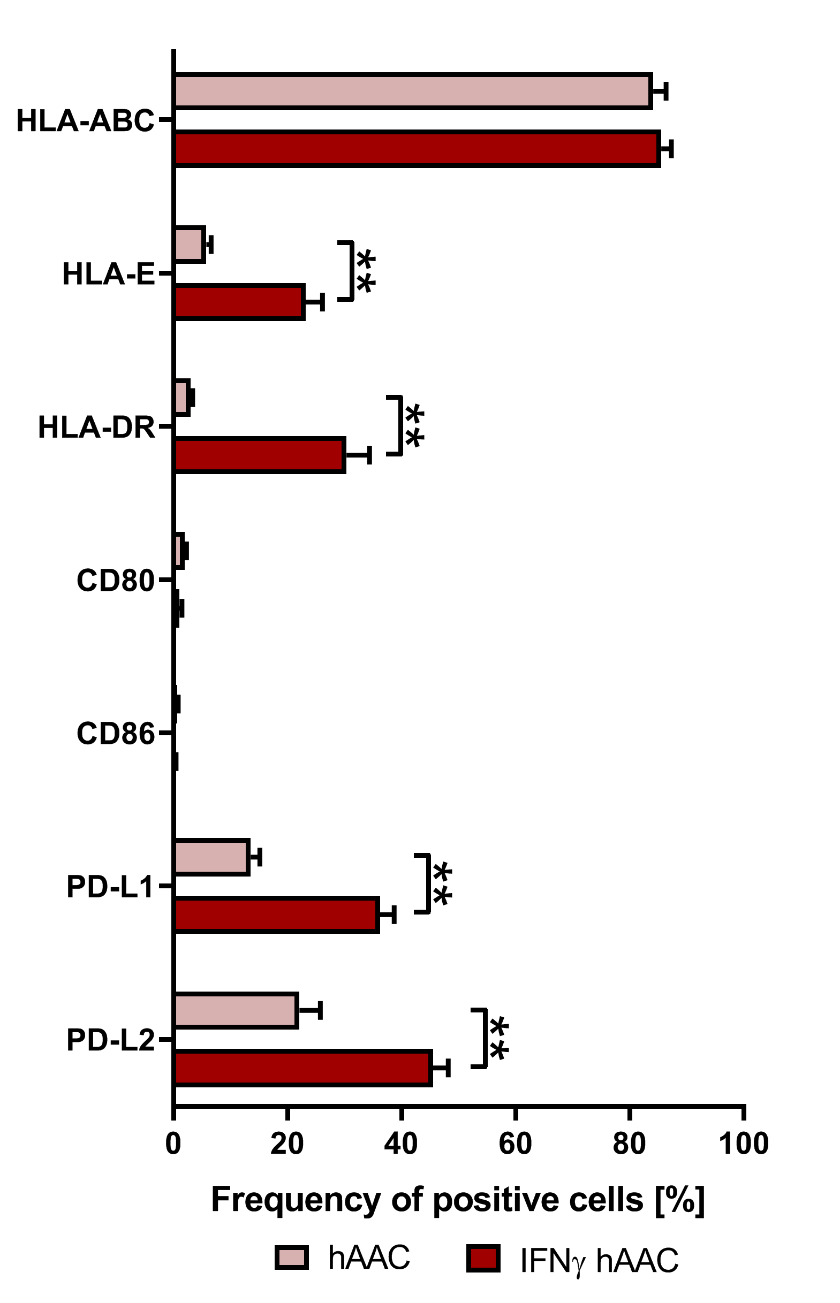


**Supplementary Figure 3:** Percentage of immune cell-marker positive hAACs in absence or presence of IFNγ pre-stimulation.

Cells were cultured for 48 hours without additional stimulation (hAAC) or in presence of 100 ng/mL human interferon-gamma (IFNγ hAAC), stained with human-specific antibodies against HLA-ABC, HLA-E, HLA-DR, CD80, CD86, PD-L1 as well as PD-L2 and were analyzed by flow cytometry. Percentage of marker positive cells are presented as mean + SEM (n = 6; three independent experiments with six different hAAC donor). Differences between hAACs and IFNγ hAACs were considered significant when ***p* ≤ *.01* with the Mann-Whitney t-test.


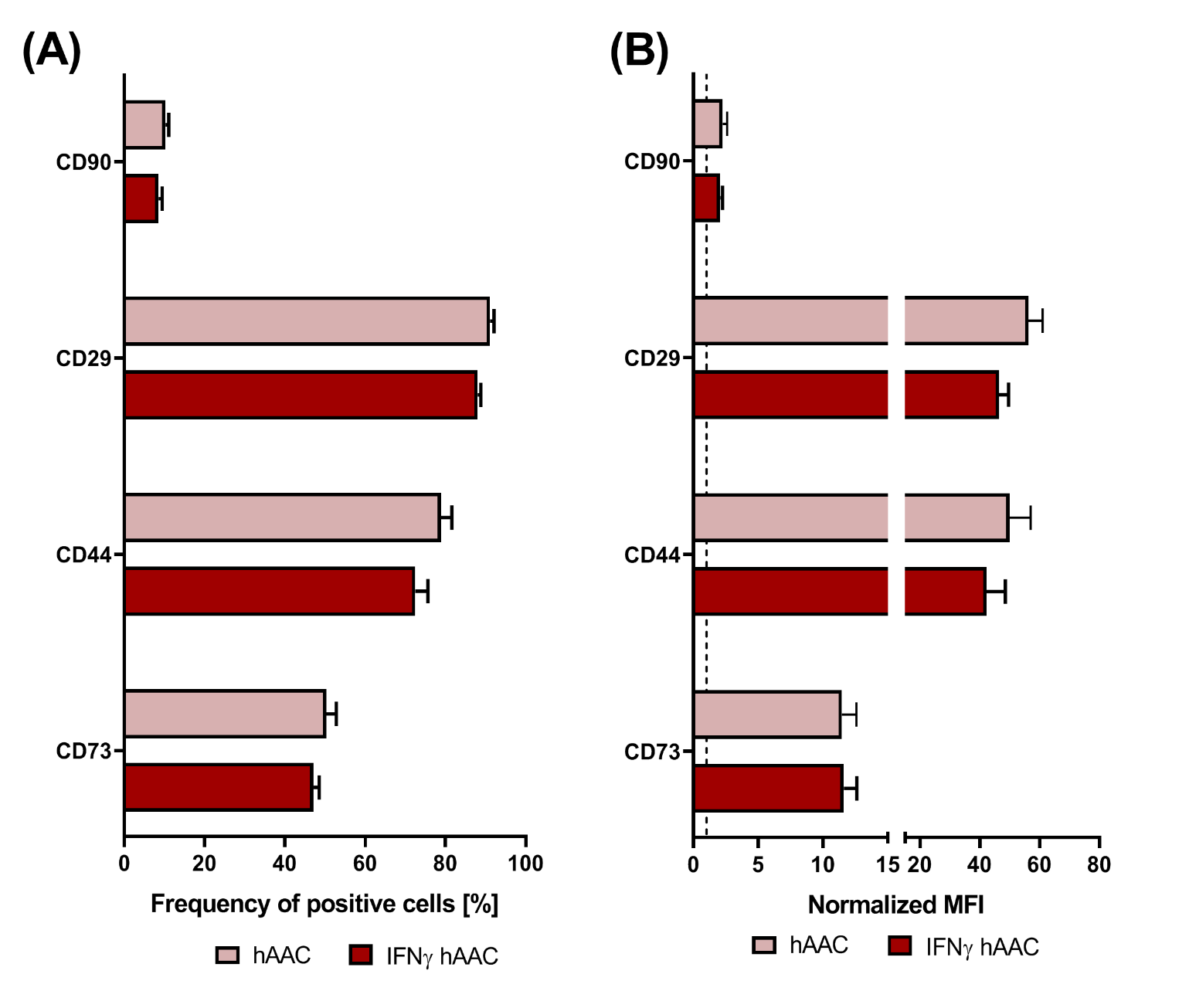
**Supplementary Figure 4:** Expression of characteristic mesenchymal stromal cell-markers on hAACs in absence or presence of IFNγ pre-stimulation.

Cells were cultured for 48 hours without additional stimulation (hAAC) or in presence of 100 ng/mL human interferon-gamma (IFNγ hAAC), stained with human-specific antibodies against CD90, CD29, CD44 as well as CD73 and were analyzed by flow cytometry. **(A)** Percentage of marker positive cells and **(B)** normalized fluorescence intensities of marker expression, that are calculated based on the respective controls (set to one; dashed black line), are presented as mean + SEM (n = 6; three independent experiments with six different hAAC donor). No significant changes were detectable between hAACs and IFNγ hAACs with the Mann-Whitney t-test: *p > .05*.


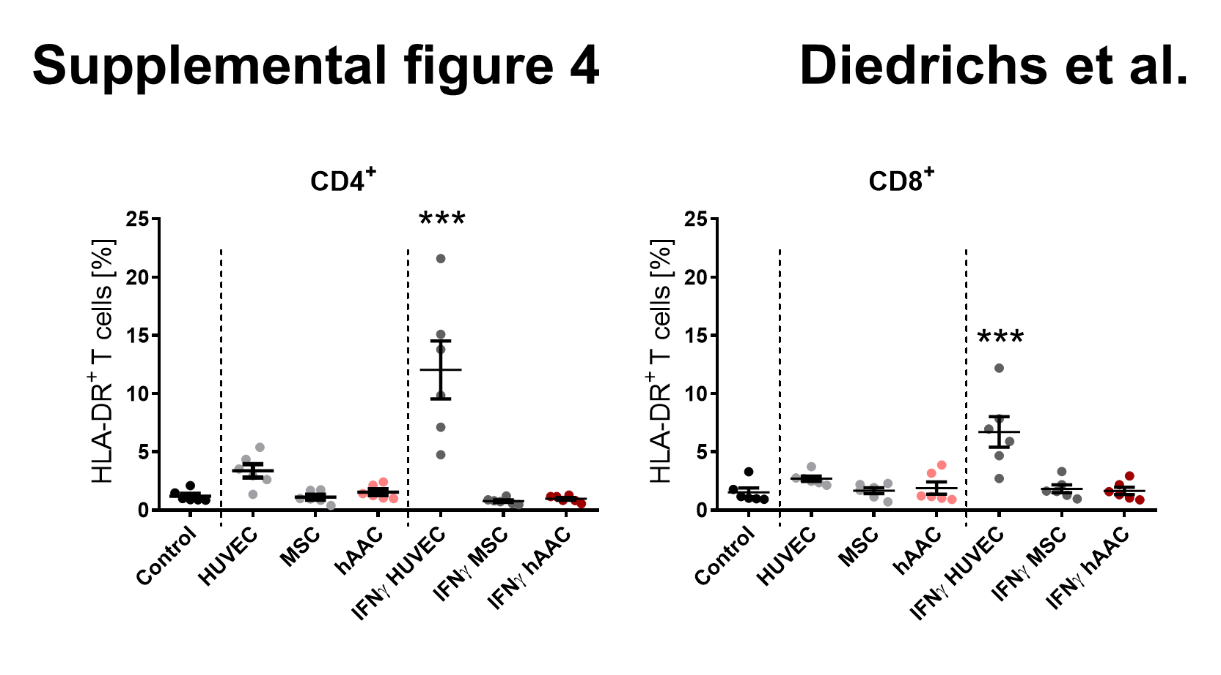
**Supplementary Figure 5:** Activation status of T cells after induced proliferation with HUVECs, MSCs and hAACs.

HUVECs, MSCs and hAACs were pre-cultured in the absence or presence of 100 ng/mL of interferon-gamma (IFNγ) for 48 hours. Following incubation with carboxyfluorescein succinimidyl ester (CFSE)-labeled, human leukocyte antigen (HLA)-mismatched peripheral blood mononuclear cells (PBMCs) for seven days, immune cells were harvested and stained for the late activation marker HLA-DR in CD4^+^ and CD8^+^ T cell subsets. Summarized data for percentages of HLA-DR^+^ T cells are presented as mean ± SEM (n = 6; three independent experiments with six different hAAC donor). Groups were considered significantly different compared to the Control (PBMCS only) when ****p* ≤ .001 with Kruskal Wallis ANOVA and Dunn’s post-test.


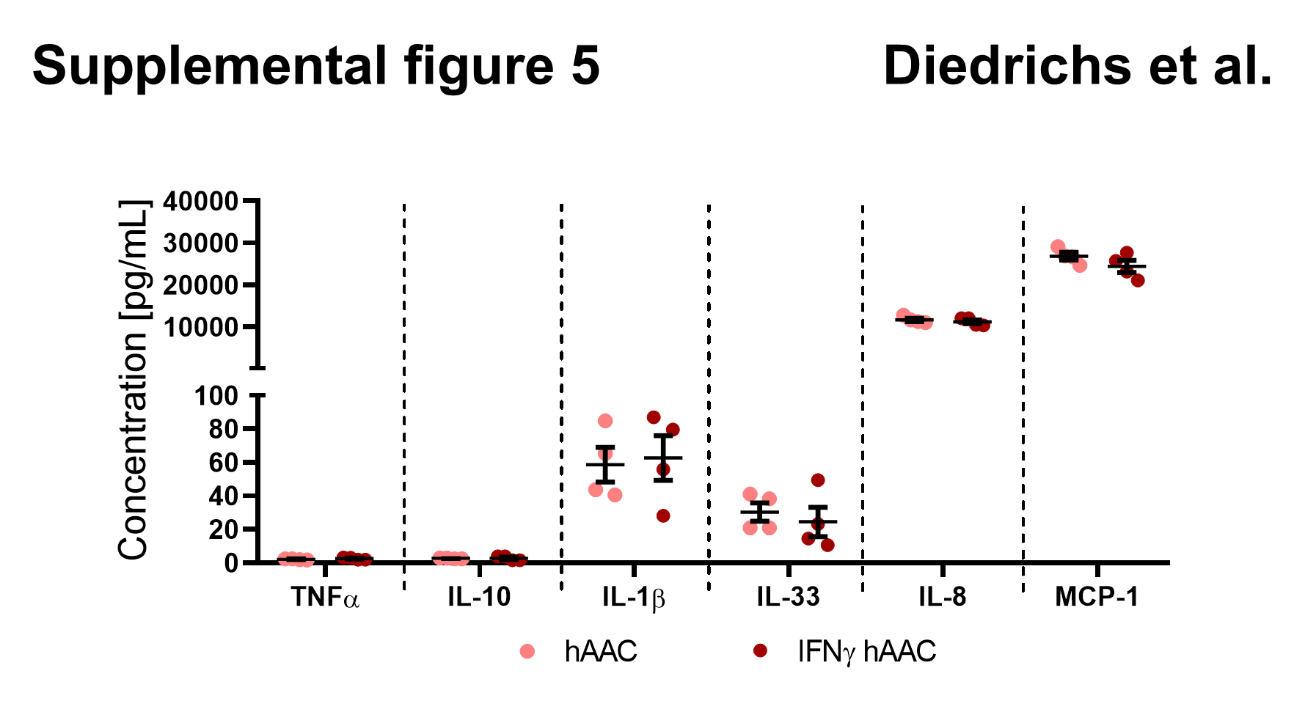
**Supplementary Figure 6:** Quantification of released cytokines from hAAC mono-cultures after four days of incubation.

hAACs were pre-cultured with or without 100 ng/mL of interferon-gamma (IFNγ) for 48 hours. Afterwards, the supernatant was discarded, and fresh medium was added to the cultures. After four days of incubation, supernatants were taken for cytokine detection using the Legendplex™ human inflammation panel. Measured cytokine levels in [pg/mL] for TNFα, IL-10, IL-1β and IL-33, IL-8 and MCP-1 are shown as mean ± SEM (n = 4; two independent experiments with four different hAAC donor). No significant changes were detectable between hAACs and IFNγ hAACs with the Mann-Whitney t-test: *p > .05*.


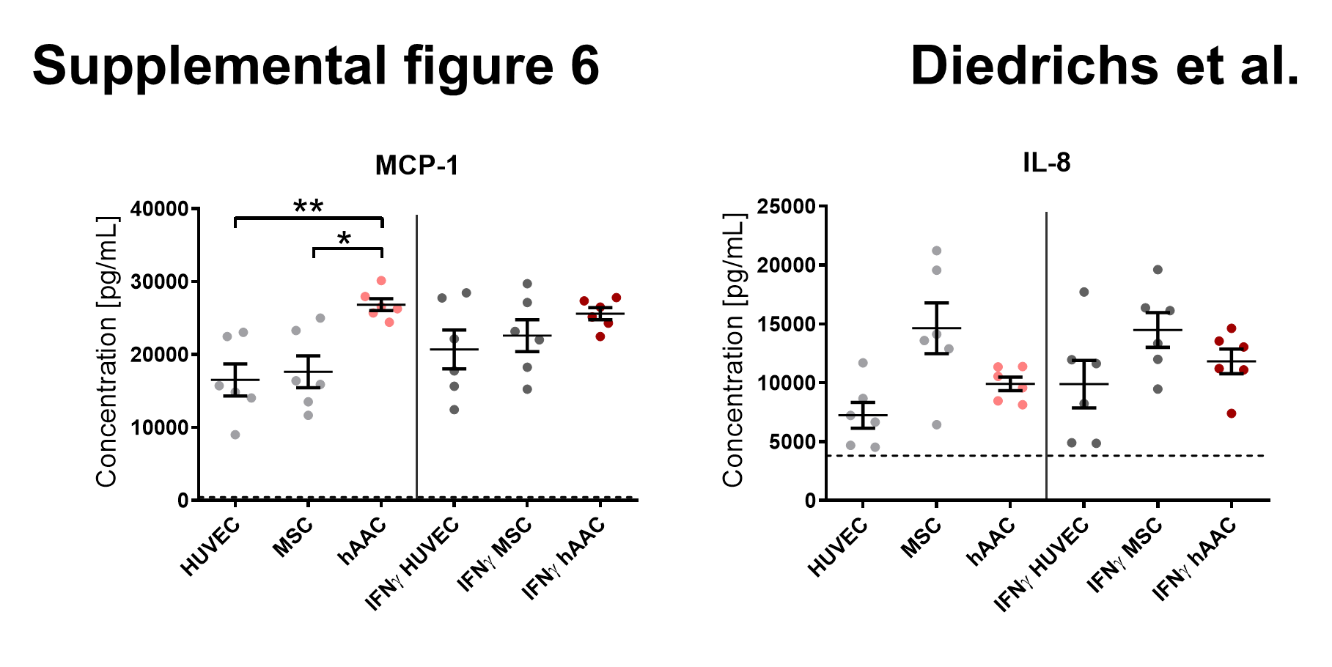
**Supplementary Figure 7:** MCP-1 and IL-8 secretion in co-cultures of induced proliferation.

Supernatants of co-cultures with CFSE-labeled, HLA-mismatched PBMCs and unstimulated or IFNγ-stimulated hAACs, MSCs or HUVECs were taken after four days of incubation and analyzed for their content of MCP-1 and IL-8 using the Legendplex™ human inflammation panel. Measured cytokine levels in [pg/mL] are shown as mean ± SEM (n = 6; three independent experiments with six different hAAC donor). Groups were considered significantly different when **p* ≤ *.05; **p* ≤ *.01* with Kruskal Wallis ANOVA and Dunn’s post-test. Differences between treatments were tested with ordinary two-way ANOVA and Sidak’s post-test: *p > .05*.


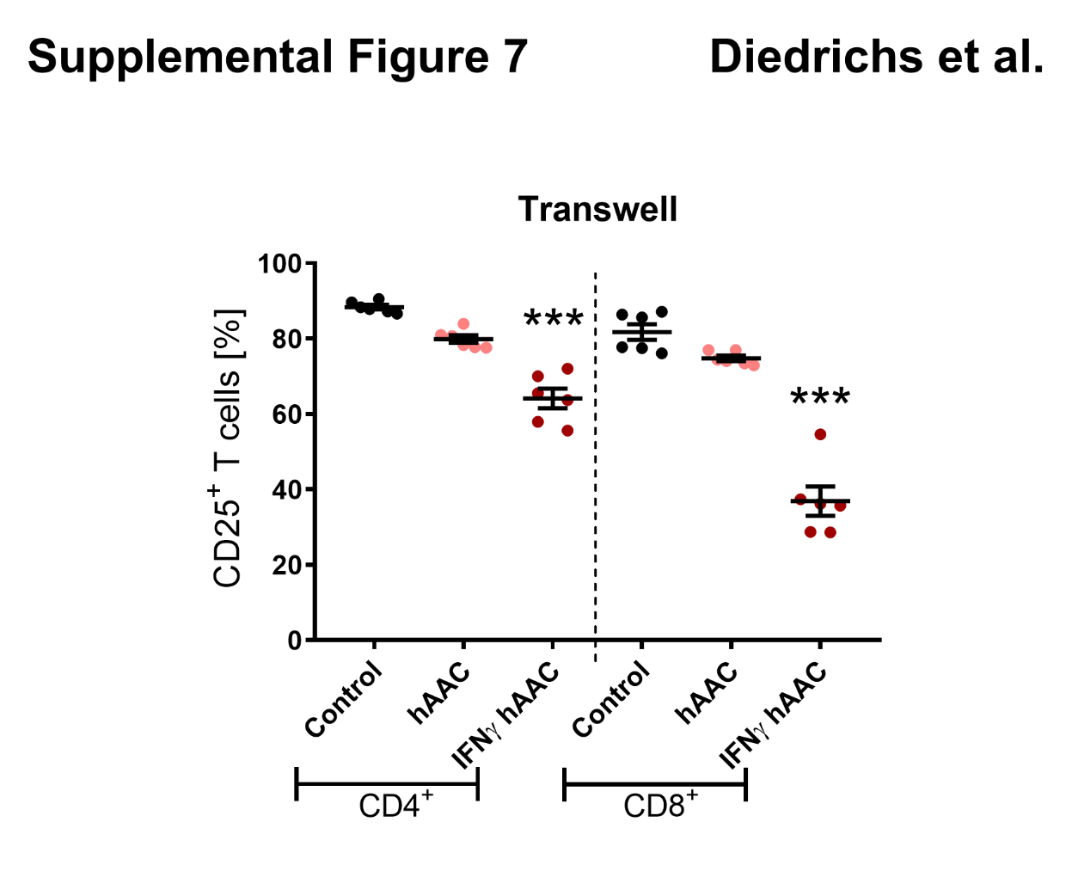
**Supplementary Figure 8:** IFNγ-stimulated hAACs diminish activation of CD4^+^ and CD8^+^ T cells in transwell settings.

CFSE-labeled PBMCs were activated with αCD3/αCD28 antibodies and cultured alone (Control) or in a transwell setting with unstimulated or IFNγ-pretreated hAACs for 72 hours. Cells were harvested, stained with human-specific antibodies and analyzed by flow cytometry for the T cell activation marker CD25 in CD4^+^ and CD8^+^ T cell subsets. Summarized data for percentages of CD25^+^ T cells are presented as mean ± SEM (n = 6; two independent experiments with six different hAAC donor). Groups were considered significantly different compared to the Control when ****p* ≤ .001 with Kruskal Wallis ANOVA and Dunn’s post-test.


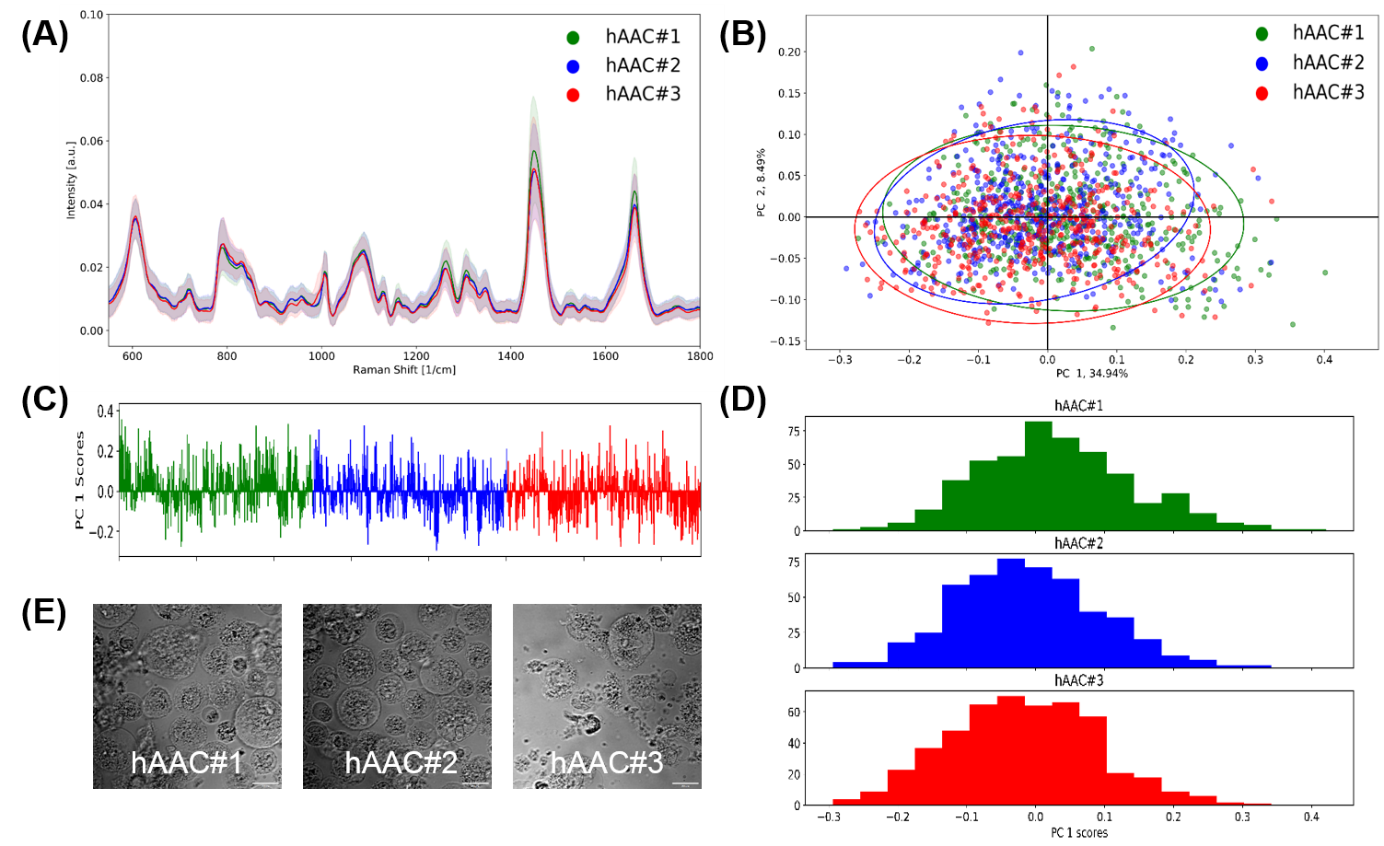
**Supplementary Figure 9:** Raman spectroscopy reveals no significant differences in the cellular composition of three hAAC donors.

**(A)** Mean Raman spectra with standard deviations of three donors. Neither 2D scores plot **(B)** nor bar plots **(C)** did reveal differences between the three donors (each dot and bar, respectively represent a measured cell). Circles in 2D scores plot depict 95% confidence intervals. These findings are supported by the histograms in **(D)**. Bright Field micrograph measurements generated with the Raman trapping microscope (CellTool GmbH, Bernried) of all three donor cells are demonstrated in **(E)**.
